# Whole genome sequencing of *M. tuberculosis* for disease control in high-burden settings: study protocol for a cluster randomized controlled trial evaluating different community-wide intervention strategies in rural Madagascar

**DOI:** 10.1186/s13063-024-08537-4

**Published:** 2024-10-25

**Authors:** Emmanuelle Sandra Adjoa Ametepe, Noela Andriamanoha, Fanantenana Randria Andrianomanana, Floriane Point, Reziky Tiandraza Mangahasimbola, Alina Dyachenko, Michael Hall, Theodora Mayouya Gamana, Astrid M. Knoblauch, Yemimah Yededyah Razafindrasoa, Arianminpathy Nimalan, Marcel Behr, Madeleine Durand, Mira Johri, Zamin Iqbal, Andry Rivo Rakotoarivelo, Rindra Vatosoa Randremanana, Niaina Rakotosamimanana, Simon Grandjean Lapierre

**Affiliations:** 1 École de Santé Publique de L’Université de Montréal (ESPUM), Université de Montréal, Montréal, Canada; 2 Centre de Recherche du Centre Hospitalier de L’Université de Montréal, Université de Montréal, Montréal, Canada; 3 Epidemiology Unit, Institut Pasteur de Madagascar, Ambohitrakely, Antananarivo, Madagascar; 4 Mycobacteria Unit, Institut Pasteur de Madagascar, Ambohitrakely, Antananarivo, Madagascar; 5 European Bioinformatics Institute (EMBL-EBI), Cambridgeshire, UK; 6 The Peter Doherty Institute for Infection and Immunity, The University of Melbourne, Melbourne, Australia; 7 Swiss Tropical and Public Health Institute, Allschwil, Switzerland; 8 University of Basel, Basel, Switzerland; 9 School of Public Health, Imperial College, London, England; 10 Research Instituteof the, McGill University Health Center , Montreal, Canada; 11 Department of Medicine, Université de Montréal, Montréal, Canada; 12 Milner Center for Evolution, University of Bath, Bath, UK; 13 Department of Microbiology, Immunology and Infectious Diseases, Université de Montréal, Montréal, Canada

**Keywords:** Tuberculosis, Whole genome sequencing, Molecular epidemiology, Disease outbreak, Cluster randomized controlled trial

## Abstract

**Background:**

Retrospective and descriptive molecular epidemiology studies have shown that *Mycobacterium tuberculosis* whole genome sequencing can identify outbreaks and disease transmission events with higher resolution than conventional epidemiological investigations. Those studies have strengthened our understanding of genomic polymorphisms correlating with person-to-person transmission and helped resolve putative transmission clusters. To date, systematic genomic surveillance programs implemented for *M. tuberculosis* were only implemented in low-incidence settings. The purpose of this study is to determine whether there is an impact of routine *M. tuberculosis* whole genome sequencing on tuberculosis case detection in a high-incidence setting.

**Methods:**

A cluster randomized controlled trial will be performed. Forty-eight rural village groups (or Fokontany) in the Vohibato district of Madagascar will be randomized to one of three interventions arms. Arm 1 (standard of care) involves healthcare facility-based passive case detection with smear microscopy testing. Arm 2 (best practice) consists of active case finding and Xpert MTB/RIF Ultra PCR testing followed by household contact investigations. Arm 3 (novel intervention) includes the same interventions as arm 2, with addition of sputum culture and *M. tuberculosis* whole genome sequencing for all newly diagnosed cases. In arm 3, molecular suggested putative outbreaks are investigated, and additional TB suspects are appropriately tested. The intervention observational period will be 2 years. The primary outcome will be the number of detected cases/100,000/year in each arm after 1 year of intervention.

**Discussion:**

This study is designed to determine whether there is an impact of prospective whole genome sequencing-based molecular typing on tuberculosis case detection in high-incidence settings. Investigating potential outbreaks and focusing active case finding in spatiotemporal settings where disease transmission is suggested by genomic typing is hypothesized to improve case detection in rural communities.

**Trial registration:**

ClinicalTrials.gov NCT05406453. Retrospectively registered on June 6, 2022.

**Supplementary Information:**

The online version contains supplementary material available at 10.1186/s13063-024-08537-4.

## Introduction

### Background and rationale {6a}

Conventional tuberculosis (TB) control interventions are predicted to fail in meeting the World Health Organization (WHO) End TB Strategy objective of eliminating TB by 2035 and the WHO calls for discovery, development, and rapid uptake of new tools and interventions [1, 2]. *Mycobacterium tuberculosis* whole genome sequencing (WGS) can detect both TB person-to-person transmission and TB drug resistance from the same clinical sample using the same diagnostic platform [3, 4]. Its use for drug susceptibility testing (DST) is driving its implementation in many countries with international TB genomics consortia support and WHO technical guidance [5–7]. *M. tuberculosis* WGS can also uncover unsuspected chains of transmission and support epidemiologic investigations beyond the usual contact tracing that is already recommended by WHO [8]. The principle behind WGS-based inference of TB transmission is that mutations, or single nucleotide polymorphisms (SNP), occur in the bacterium over time; if two isolates are very related or divergent (below or above the number of SNPs expected for a given period of time), transmission may respectively be suggested or refuted [9]. Retrospective and descriptive molecular epidemiology studies have shown that *M. tuberculosis* WGS can identify person-to-person transmission events with higher resolution than previous molecular typing methods [10]. To date, no data has showed that prospective and systematic sequencing favorably impacts TB case detection or limits the spread of disease. The hypothesis that prospective recognition of transmission events could lead to diagnosis and treatment of additional patients and further limit transmission remains to be proven. TB transmission is impacted by community social, spatial, and cultural dynamics [11]. In high-incidence settings, TB control strategies are designed and implemented through programmatic planning by public health authorities. In this context, a cluster randomized control trial study design comparing different intervention bundle strategies in distinct but comparable communities is well adapted to assessing the value of prospective TB molecular epidemiology for disease control within communities.

Madagascar exemplifies the challenges of TB control in remote and low-resource settings as the country only diagnoses 61% of its cases [12]. Access to TB diagnosis was shown to represent the most important gap in the Malagasy TB care cascade [13]. On the other hand, incidence of HIV and multi-drug resistant tuberculosis (MDR-TB) are low and the majority of the population is rural, isolated, and geographically stable [13, 14]. These conditions offer a unique opportunity to achieve substantial progress towards TB elimination if case detection is significantly improved and transmission interrupted. The country’s National Tuberculosis Program reference laboratory at the Institut Pasteur de Madagascar has recently implemented TB WGS-based diagnostics as a research use only assay for surveillance and DST [15]. Madagascar is hence a well-adapted setting to assess the impact of molecular typing on disease control in high-incidence settings.

## Objectives {7}

The study objective is to measure whether there is an impact of systematic and prospective *M. tuberculosis* WGS on TB case detection in high-incidence settings. We hypothesize that WGS-guided epidemiologic investigations improve case detection by adapting contact tracing strategies to local transmission patterns.

## Trial design {8}

This factorial, three-arm, open cluster randomized controlled trial (cRCT) will compare three TB control interventions in the Vohibato rural district of Madagascar. In rural Madagascar, villages of ~200 people include ~20 households and are administratively grouped in Fokontany (smallest administrative unit), of ~9 geographically close villages”. Fokontany (“1800 inhabitants”) will be the unit of randomization and analysis for this trial. Fokontany satisfying inclusion/exclusion criteria (eligibility criteria) will be selected and 48 out of them will be randomized by minimization to three equal arms [16].

## Methods: participants, interventions, and outcomes

### Study setting {9}

The study will take place in Vohibato district located in the Haute Matsiatra region in Madagascar. The district of Vohibato includes 232,518 inhabitants and is subdivided in 14 communes and 145 Fokontany [17]. Field case finding and patient recruitment activities will be deployed in rural Fokontany (i.e., Malagasy administrative entity consisting of an aggregation of villages). Local public TB CDT will perform primary diagnostic assays. The National Tuberculosis Program (NTP) reference laboratory hosted at the Institut Pasteur de Madagascar (IPM) will perform reference testing including bacterial culture and sequencing.

### Eligibility criteria {10}

There are 145 Fokontany in Vohibato. Of those, 132 were categorized as rural in this study because of their location associated with limited access to healthcare facilities and urban areas. Those 132 Fokontany correspond to the target study population. Fokontany with at least one active TB case confirmed during the 6-month period prior to randomization will be eligible. Fokontany further than 100 km from a primary care TB clinic or that elicit safety concerns or geographic accessibility issues will not be included. There are 56 Fokontany which fulfill those eligibility criteria. Out of those, 48 will be allocated in three equal arms using randomization by minimization (see “ Assignment of interventions: allocation” section).

### Who will take informed consent? {26a}

Since TB screening and laboratory testing of symptomatic patients are recommended as per NTP guidelines and represent the standard of care, the need for informed consent will be waived for inhabitants of Fokontany randomized to arm 1. Written informed consent will only be obtained upon confirmation of a TB diagnosis for patients living in Fokontany randomized to arm 2 and arm 3, prior to conducting sputum culture for genomic DNA sequencing and outbreak investigation which are not part of standard of care. Clinical research personnel with up-to-date training in good clinical practices (GCP) will obtain participants informed written consent before inclusion. The study consent form will be available to participants in French and Malagasy and is available as Supplementary Materials 2. The consenting process will follow local ethics guidelines from the local ethics board. Should patients refuse to participate in the study, they will still have access to the same standard of care, as per local NTP guidelines. Participants will be able to withdraw from the study and request that their data be deleted at any time. Amendments to the study protocol will be required and re-consenting will be required for collection and use of additional participant data or biological specimens.

### Additional consent provisions for collection and use of participant data and biological specimens {26b}

N/a: We will not conduct any ancillary studies.

## Interventions

### Explanation for the choice of comparators {6b}

Arm 1 “standard of care” will provide a country contextualized baseline for TB diagnosis and notifications which reflects the current NTP strategy. Arm 2 “best practice” will provide a measure of how well current recommendations including WHO guidance perform against the study intervention in the specific study context. Data from arm 2 will also allow to assess whether perceived added value of WGS is driven by the impact of diagnostic (Xpert MTB/RIF Ultra PCR) sensitivity or active rather than passive case finding.

### Intervention description {11a}

**Fig. 1:**
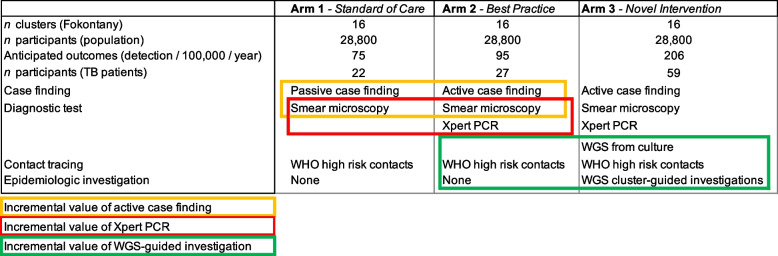
cRCT study arms, sample size, anticipated outcomes, and intervention strategies

Case finding strategies are either passive—meaning health facility-based and limited to self-presenting patients—or active—meaning health system-initiated and targeting patients beyond healthcare infrastructures. TB diagnosis either relies on smear microscopy (sensitivity 63%) or polymerase chain reaction (PCR) molecular testing (sensitivity 89%) [18–21]. Contact tracing activities prioritize high-risk household contacts [9]. Clusters will be randomized into one of 3 arms, all integrating a bundle of case finding, laboratory diagnosis, and contact tracing strategies (see Fig. 1). Arm 1 will represent the high-burden low- and middle-income countries (LMIC) “standard of care” and include passive case finding, smear microscopy, and household contact tracing. Arm 2 will represent WHO recommended “best practice” and additionally include Xpert PCR MTB/RIF Ultra and active case finding in which clinical teams will visit Fokontany to seek out and test individuals presenting TB-compatible symptoms. Arm 3 will represent the “novel intervention” which, in addition to interventions included in arm 2, will add sputum culture, *M. tuberculosis* WGS and epidemiologic investigations to detect additional TB cases. *M. tuberculosis* WGS will be performed using already implemented Oxford Nanopore Technologies™ [15]. The offline or cloud-based TB bioinformatic analysis pipeline TB Pore Cluster will be used for isolates clustering and transmission detection. SNP transmission thresholds (5–12 SNPs) were previously reported from low-incidence countries [22]. To avoid the overlook of transmission events and maximize TB detection, a minimally stringent threshold (12 SNPs) will be used to trigger field epidemiologic investigations. When molecular typing suggests patients clustering, field clinical teams will investigate epidemiologic correlates of transmission events such as co-farming and co-attendance to school or service institutions. If such are present, additional contact tracing and testing based on newly identified epidemiologic denominators between related patients will be performed.

### Criteria for discontinuing or modifying allocated interventions {11b}

Interventions will be implemented at healthcare system level. No compliance issues or loss to follow-up of clusters are anticipated. No modification to the allocation or interventions in included Fokontany is planned regardless of the interim analyses study results.

### Strategies to improve adherence to interventions {11c}

To ensure high rates of initial consent and retention in the study during local investigation, consultation with, and consenting from, community leaders and health officials will be sought. Comprehensive study information will also be provided in Malagasy to participants.

### Relevant concomitant care permitted or prohibited during the trial {11d}

No TB-related non-governmental organization or novel country-initiated interventions are planned in the study area.

### Provisions for post-trial care {30}

All TB patients will be referred to NTP for free access treatment and clinical follow-up. This study involves no risk for the safety of participants.

### Outcomes {12}

Globally, TB incidence is reported as number of cases/100,000 population/year. TB cases are defined by WHO as pulmonary or extra-pulmonary TB, adult or pediatric TB, and bacteriologically confirmed or non-confirmed TB. Within underdeveloped health systems, number of cases diagnosed by NTPs (case detection/100,000/year) is usually less than real incidence as estimated by WHO. Case detection/100,000/year is a standardized WHO indicator allowing comparisons between interventions. It is also the best available surrogate marker for impact on transmission and short- and long-term disease control. In this study alike, the primary outcome will be the difference in TB incidence (TB case detection/100,000/year) between arms 3 and 2 during the first year of the study intervention. Secondary outcomes will include the difference in TB incidence between arms 3 and 1 and between arms 2 and 1. Secondary outcomes will also include all the above incidence difference between arms during 2 years of intervention. For all primary and secondary outcomes, sub-group analyses based on biological sex, age, and baseline cluster characteristics will also be performed. Generally, an increase in TB detection is desirable since additionally diagnosed patients would otherwise go unnoticed, transmit, and either self-present to care at a later stage of disease or die. In this study, the objective will not be to accurately assess disease incidence but rather to measure the increase in the primary outcome between arms.

### Participant timeline {13}

Active case finding and associated laboratory testing will be performed every year in arms 2 and 3 clusters. Study interventions and data capture will last 2 years for all clusters.

### Sample size {14}

**Fig. 2:**
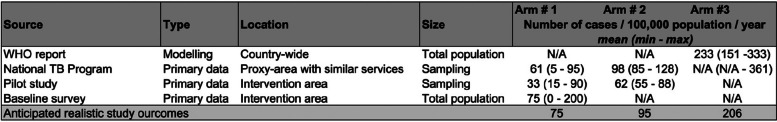
cRCT anticipated outcomes, data sources, and assumptions

This study will be powered to demonstrate superiority of arm 3 “novel intervention” over arm 2 “best practice” after 1 year. Given the purely incremental nature of arm 3 “novel intervention,” it is assumed it cannot be inferior. Using (i) realistic anticipated outcomes described below with 116% increase in TB case detection rate from 95/100,000/year to 206/100,000/year, (ii) Fokontany as the analysis unit, (iii) intraclass correlation coefficient (ICC) of 0.0005 for sample size adjustment, (iv) average Fokontany cluster size of 1800 inhabitants, and (v) assuming a one-sided *α* = 0.05 test with 80% power, we estimate the number of Fokontany in each arms should be 16 to allow detection of the TB detection rate difference using a simple *t*-test weighed by Fokontany size [23]. TB incidence data from every Fokontany in the Haute Matsiatra region were used to perform sample size calculation. Given the sufficient number of eligible Fokontany in the Vohibato district, 48 Fokontany from this district will be selected to increase feasibility and cluster homogeneity. Multiple simulations were performed to strengthen this sampling size strategy and are presented as Supplementary Materials 1. To estimate anticipated outcomes in each arm, to calculate the ICC and calculate sample size, we used (i) Madagascar TB incidence estimates from WHO, (ii) NTP data disaggregated by primary healthcare facility where services are similar to study interventions, (iii) a comprehensive and systematic baseline survey, and (iv) pilot studies assessing clusters interconnections and impact of arm 1 and arm 2 interventions (see Fig. 2) [11]. Using demographic, distance to healthcare facility, and baseline TB detection rates, the ICC was calculated to be 0.0005. This suggests homogeneity between clusters, which agrees with findings of social network studies performed in the same area and ICC values used in sub-Saharan African studies measuring TB case finding strategies in similar contexts [24]. For arm 1 “standard of care,” anticipated outcomes are adjusted to most reliable, contextualized, and comprehensive primary data from the baseline survey, 75/100,000/year. Using all four sources of preliminary data, we determined realistic anticipated outcomes for arm 2 to be 95/100,000/year and 206/100,000/year for arm 3 [11, 12, 14].

### Recruitment {15}

Clusters will be included at a rate of ~2–3 per month per arm. Based on successes in the pilot study (6 Fokontany per month and community acceptability was high without TB-related stigma impeding activities), this enrolment rate is highly feasible.

## Assignment of interventions: allocation

Fokontany characteristics that were likely to be associated to the primary outcome of TB detection rates were identified as potential confounders. Based on results of a pilot study from the same rural area, those include (i) the baseline TB notification rate in the year prior the beginning of the trial, (ii) distance to the closest TB diagnostic and treatment center (CDT), (iii) population in each Fokontany, and (iv) closest TB care center. These continuous variables will be dichotomized using median measures for eligible Fokontany only. All these four factors will be included into the randomization algorithm, which will be done by minimization with equal treatment allocation [16, 25]. The allocation by minimization will decrease the risk of substantial imbalance in those important factors. The total imbalance for each treatment is calculated by summing the weighted imbalance scores across all prognostic factors [26]. The pre-specified covariate weights of 0.3, 0.3, 0.3, and 0.1 will be applied for baseline TB incidence, distance to CDT, Fokontany population, and closest TB care center covariates respectively. The weights were chosen based on the covariates’ distribution and potential clinical judgment. Fokontany will be randomized with an allocation ratio of 1:1:1 to assign 16 Fokontany clusters in each of arms 1, 2, and 3. Allocation by minimization with equal treatment allocation will be performed using R version 4.4.1.

### Sequence generation {16a}

Cluster allocation by minimization of 48 out of 145 Fokontany in arm 1, 2, or 3 will be performed by statistician prior the beginning of intervention. The covariates distribution by study arm will be reported. The intervention implementation order will be randomly and uniformly generated across quarters during the first year.

### Concealment mechanism {16b}

N/a: Concealment is not required since allocation will be performed randomly using statistical software.

### Implementation {16c}

Inclusion, stratification, and randomization will be overseen by a biostatistician. Field clinical teams will enroll participants.

## Assignment of interventions: blinding

### Who will be blinded {17a}

This will be an open-label trial. The potential undesired effect of the open-label design is that of patients seeking care in Fokontany randomized to different arms to access better diagnostic testing. With the “intention to treat” analysis, such detected cases would be recorded in their respective cluster and tend to reduce apparent impact of the novel intervention. Contamination between clusters is not expected because (i) rural Madagascar sociodemographic structure is such that populations are stable within their home Fokontany, (ii) a social network analysis (SNA) pilot study suggests almost absent interactions between persons from different Fokontany (pilot study), (iii) distance between clusters averages 6 km of twisting footpaths, and (iv) out of 145 Fokontany in the study area, 48 will be included.

### Procedure for unblinding if needed {17b}

N/a: as explained above, our study did not involve any blinding.

## Data collection and management

### Plans for assessment and collection of outcomes {18a}

TB case detection will be prospectively recorded in local CDTs. Data will be cross validated with both study records and NTP centrally aggregated data on a quarterly basis.

### Plans to promote participant retention and complete follow-up {18b}

Interventions will be implemented at healthcare system level; no participant retention or follow-up will be required.

### Data management {19}

Data collection and entry will be conducted by the study nurse. Data will be entered directly into a tablet and sent daily to the IPM server for storage. The data manager team will perform the data quality process, and data queries will be sent to the field team daily. All data will be stored on the IPM server, with a recovery system in place to protect against data loss. The main server is duplicated at a separate location. The study’s data manager will oversee the data management SOPs.

### Confidentiality {27}

All participants will be given a unique identifier (ID). Study results dissemination mechanisms will not allow for direct or indirect identification of patients. All information will be anonymized for downstream analyses including those which require inter-institutional data transfer between the investigators.

### Plans for collection, laboratory evaluation, and storage of biological specimens for genetic or molecular analysis in this trial/future use {33}

Clinical samples will be collected within communities during active case finding activities. Smear microscopy and Xpert MTB/RIF Ultra PCR testing will be performed at the Tambohobe regional hospital. Samples positive for TB will be referred to IPM reference laboratory for culture and WGS testing, results analysis, and reporting. Cultured bacterial strains will be stored at least 15 years at IPM. Primary clinical samples will not be stored.

## Statistical methods

### Statistical methods for primary and secondary outcomes {20a}

First, we will calculate TB case notification rates for each Fokontany. The overlapping TB cases resulting from our RCT study and from NTP report will be counted once in the outcome measures. The primary analysis will compare notification rates between arms after 1 year, using a simple *t*-test and nonparametric Mann–Whitney *U* test weighed by Fokontany size with Fokontany as the unit of analysis. Detection rate differences and 95% confidence intervals (CI) will be reported. The ICC will be calculated from the output of one-way analysis of variance (ANOVA) [27]. As a secondary analysis, the longer-term effect of sustained interventions will be evaluated for 2 years. The longitudinal impact of interventions will be evaluated with a linear mixed-effects model weighed by Fokontany size again conducted at the cluster level. The model will include the yearly TB detection rate, the study arm (1, 2, and 3), the year (first and second), and the interaction arm × year as the fixed effects and Fokontany as the random effect. The least squares mean estimates with 95%CI will be computed from the models. The longitudinal pattern of TB detection rate for each study arm and for each Fokontany will be presented graphically by 6- and 12-month interval. To estimate Fokontany size, we will use two approaches. First, we will consider primary data from the 2019 INSTAT-World Bank census (pilot study) with the 2.7% World Bank estimated annual increase [28, 29]. Then, we will consider the population reported by health authorities upon case finding visits. R software will be used for analyses.

### Interim analyses {21b}

Interim results will be analyzed every 12 months by the steering committee. If a statistically significant benefit is shown early in the study, results will be disseminated, and the study will be continued until interventions have been implemented for a 2-year period in all clusters to assess longer term impact on TB control (statistical methods for primary and secondary outcomes). If no significant difference between groups is seen after 1 year, this study will be considered negative. We will complete 2 years of intervention to further investigate transmission risk factors emerging from outbreak investigations and long-term impact on case detection rates.

### Methods for additional analyses (e.g., subgroup analyses) {20b}

This study is not powered for sub-group analyses. We will report outcomes stratified (i) by available Fokontany level covariates (baseline TB rate, distance to the closest CDT, Fokontany population in each Fokontany, and closest NTP center) and (ii) by collected demographic variables of TB case participants (sex: female vs male). The exact demographic measures for non-TB case habitants are non-known and will be assumed of (i) equal sex proportions and (ii) 14.4% children < 5 year old age proportion [18]. This analysis will inform on the added value of epidemiologic investigations in women among which TB is frequently under-reported [30, 31].

### Methods in analysis to handle protocol non-adherence and any statistical methods to handle missing data {20c}

The unit of analysis is the Fokontany, and the intervention is at community level. Since we will use administrative quarterly reported data from local CDTs, we do not expect to have missing values for primary outcomes and cluster level covariates. Any inconsistencies for the individual level covariates (sex, age < 5 years) will be investigated with health authorities responsible for data collection. If the values cannot be verified, the simple imputation will be performed. The source of TB case detection for arms 2 and 3 (active case finding or standard of care) will be collected and reported descriptively by each calendar quarter.

### Plans to give access to the full protocol, participant-level data, and statistical code {31c}

The full study protocol and research tools are included in this publication and supplementary materials.

## Oversight and monitoring

### Composition of the coordinating center and trial steering committee {5d}

A trial steering and data safety committee will meet every 12 months and will include the principal investigator (PI), a biostatistician, the head of the local reference laboratory, the head of the in-country institution epidemiology unit, and external experts in TB molecular epidemiology and genomics. Its role will be to review aggregated results and compliance to the protocol as well as to periodically re-evaluate the need to amend study objectives and procedures. A local knowledge translation committee will meet every 6 months and will include regional health officials and community representatives. Its role will be to maintain communication between researchers and the study communities as well as providing feedback on results to local institutions.

### Composition of the trial steering committee, its role and reporting structure {21a}

A trial steering committee will meet weekly and will include the PI, the study coordinator, the data manager, and in-country co-investigators. Its role will be to address routine challenges in study implementation and ensure fluid communication between laboratory and clinical partners. The trial steering committee is independent from the sponsor. The sponsor (CIHR) is a Canadian public funding agency that plays no role in study design, activities, or results reporting.

### Adverse event reporting and harms {22}

This trial strictly involves case finding, diagnosis, and contact tracing. There are no safety or health risks to participants in this study as there is no additional therapeutic intervention or clinical sampling required. TB patients are referred to the NTP for free access to treatment and follow-up.

### Frequency and plans for auditing trial conduct {23}

A weekly audit of data entry will be performed by the local coordinator and local data manager. A trimestral field site audit will be performed by the institution’s clinical trial expert. No external audit will occur.

### Plans for communicating important protocol amendments to relevant parties (e.g., trial participants, ethical committees) {25}

Important modifications to the protocol will be communicated to relevant parties, including the central operational team, the local and Canadian Institutional Review Board (IRB), the NTP, and the trial registries. Local officials, responsible for patients’ treatment and follow-up, will also be notified.

## Dissemination plans {31a}

For all dissemination activities, authorship will follow guidelines of the International Committee of Medical Journal Editors (ICMJE). We aim to publish study results in international scientific conferences and peer-reviewed publications. Results will be presented at the Malagasy Infectious Diseases Society (SPIM) annual conference. Results will be presented to local health officials and community representatives in regional meetings.

## Discussion

This trial aims to prove systematic prospective WGS embedded within TB control programs in high-incidence settings allows to identify transmission chains and leads to additional diagnoses and patients’ notifications. Although several countries or public health agencies have transitioned to systematic WGS for TB (Netherlands, England, New York State), those are low-incidence settings where transmission dynamics are expected to be different and the measured impact on TB control cannot be assumed to be the same in countries like Madagascar.

## Trial status

**Fig. 3:**
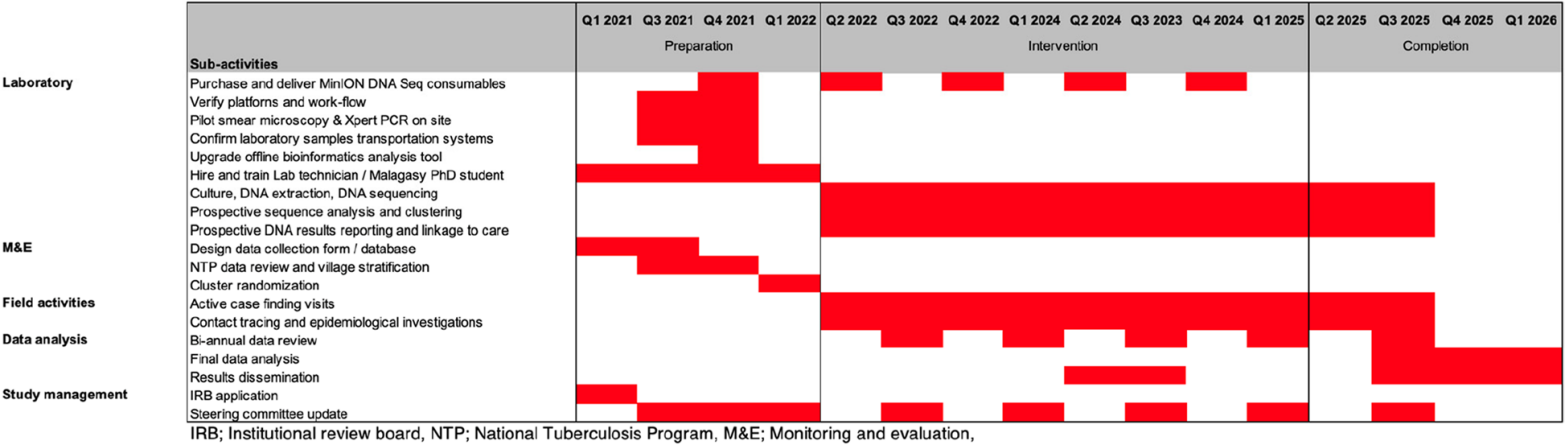
Schedule of enrolment, interventions, and assessment SPIRIT figure

The trial with protocol version 3.0 (January 28, 2022) was registered on the ClinicalTrials.gov register with the number NCT05406453 on June 10, 2022. Inclusion started on May 16, 2022, and is expected to be completed by the end of September 2025 (see Fig. 3).

## Supplementary Information

Supplementary Material 1.

Supplementary Material 2.

Supplementary Material 3.

Supplementary Material 4.

## Data Availability

The final trial data will be available upon publication of the study result. Additional data will be available upon request to the corresponding author. Researchers are not limited to any contractual agreements that limit data accessibility.

## Abbreviations

CERBM: Comité d’Éthique à la Recherche Biomédicale de Madagascar
cRCT: Cluster randomized controlled trial
DST: Drug susceptibility testing
GCP: Good Clinical Practices
HIV: Human immunodeficiency virus
ICC: Intraclass correlation coefficient
ID: Identifier
IRB: Institutional Review Board
MDR: Multi-drug resistant
NTP: National Tuberculosis Program
PCR: Polymerase chain reaction
PI: Principal investigator
SAS: Statistical analysis system
SNP: Single nucleotide polymorphism
TB: Tuberculosis
WGS: Whole genome sequencing
WHO: World Health Organization
