# Supplementary material for: Whole genome sequencing of M. tuberculosis for disease control in high-burden settings: study protocol for a cluster randomized controlled trial evaluating different community-wide intervention strategies in rural Madagascar
Source: Trials. 2024 Oct 25;25:717. doi: 10.1186/s13063-024-08537-4 (PMC11515128; doi:10.1186/s13063-024-08537-4)
Supplement: Supplementary file 1 — Supplementary Material 1. [file 13063_2024_8537_MOESM1_ESM.pdf]

Figure 1 - Sample size estimation using simulations

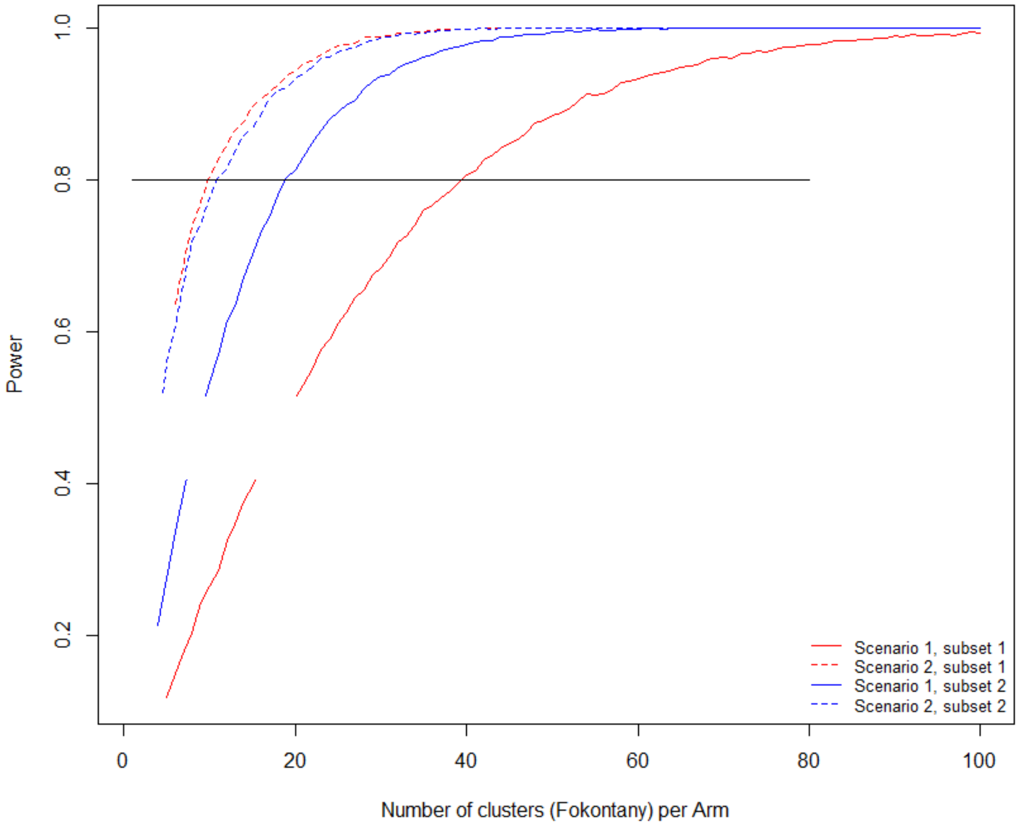

Figure 1 presents the power as the function of the number of Fokontany in each study arms.

We have developed a simulation based on simple t-test weighed by Fokontany size with Fokontany as the unit of analysis. To take in account different cluster size all 730 rural Fokontany with no more than 100km distance from health center were considered in the analysis (*Subset 1*). TB case detection for Arm1, with the average value 75/100,000/year, was provided by primary care TB clinics in the study area. First, we approximated each Fokontany TB case detection rate (i) with the constant relative risk of 1.27 (=95/75) and 2.75 (=206/75) for Arm 2 and Arm 3 comparing to Arm1 (*Scenario 1*) and (ii) with the constant difference in the TB detection rate of 20/100,000/year (=95/100,000/year -75/100,000/year) and 131/100,000/year (=206/100,000/year -75/100,000/year) for Arm 2 and Arm 3 comparing to Arm1 (*Scenario 2*). Second, the same fixed number (between 4 and 80) of Fokontany was randomly selected for each Arm. Third, we fitted a t-test weighted by cluster size for selected Fokontany to compare TB detection rate between Arm 2 and Arm3. The significance was evaluated at Alpha =0.05, one-sided test. Finally, the power was estimated after 10000 independent iterations.

To increase TB case detection rates in all arms, the subset of 437 Fokontany with at least one TB case (*Subset 2*) were considered. In addition, the analysis was repeated with the balanced stratification described in *2.3 Allocation of participants.* The result was presented in the graphical form. All calculations were performed in R version 4.0.3.

The power 80% corresponds the sample size of 39 and 10 Fokontany per arm for *Scenarios 1 et 2* respectively in Subset 1. These sample sizes are reduced to 19 and 11 Fokontany per arm if only Fokontany in *Subset 2* are considered. The simulations with and without stratification provided the same estimations of power however the smaller standard errors of power estimates were found for stratified simulations (results not shown).
