# Supplementary material for: Whole genome sequencing of M. tuberculosis for disease control in high-burden settings: study protocol for a cluster randomized controlled trial evaluating different community-wide intervention strategies in rural Madagascar
Source: Trials. 2024 Oct 25;25:717. doi: 10.1186/s13063-024-08537-4 (PMC11515128; doi:10.1186/s13063-024-08537-4)
Supplement: Supplementary file 2 — Supplementary Material 2. [file 13063_2024_8537_MOESM2_ESM.pdf]

Je soussigné(e) :

- Nom :……………………………………………………………………………………
- Prénoms :………………………………………………………………………………..
- Age : |__|__| ans
- Genre : ◯Masculin ◯Féminin
- PERS : |__|__|__|__|__|
- DE / ID / EP : |__|__|__|__|__|

Atteste par la présente ma volonté réelle de participer à ce projet de recherche intitulé **« Séquençage du Génome Complet de la Tuberculose pour le Contrôle de la Maladie à Madagascar - Un Essai Contrôlé Randomisé en Grappe pour Évaluer Différentes Stratégies d'Intervention à l'Échelle Communautaire** » en collaboration avec l'Institut Pasteur de Madagascar, l'Hôpital Universitaire de Fianarantsoa et Centre de recherche du Centre hospitalier de l'Université de Montréal, au Canada et financé par les Instituts de Recherche en Santé de Canada (IRSC).

❖ J'ai bien lu et compris toutes les informations à savoir que l'on m'a données concernant cette étude dont :

- Les objectifs et le contexte dans lequel s'inscrit l'étude
- La raison pour laquelle on m'invite à y participer
- Les méthodes et procédures de l'étude
- Les examens et suivi de santé des participants
- Les avantages et les risques relatifs à la participation ainsi que les parts de responsabilité du promoteur
- Le respect de l'éthique vis-à-vis des participants (confidentialité, sécurité, anonymat, …)
- Les compensations éventuelles
- Les réponses claires aux questionnements que le participant s'est posées et les compléments d'informations éventuels
- Les contacts et adresse où joindre l'investigateur principal ou son délégué

❖ J'accepte que les données me concernant dans l'étude puissent être vues et utilisées par les responsables de la recherche ainsi que les autorités de santé pour d'autre recherche que ce soit au niveau local qu'à l'extérieur si besoin.

- J'accepte de donner un échantillon de crachat pour servir à l'étude et conservation au niveau du laboratoire de l'Institut Pasteur de Madagascar (IPM).
- Je comprends que ma participation à l'étude est totalement volontaire et libre et que cela ne fait en aucun l'objet de rémunération
- On m'a donné le choix, sans aucune contrainte, et je pourrai à tout moment me retirer de l'étude.
- On me donne une copie de ce consentement, une copie restera auprès de l'investigateur
- Après discussion, entre moi et l'investigateur, et que j'ai obtenu les réponses à mes questions, j'accepte volontairement et librement à participer à l'étude.

Votre signature documente votre consentement à participer à cette étude

**Nom de la personne participante**	**Signature ou empreinte digitale**	**Date**

Si la personne ne sait pas lire ni écrire :

Un témoin qui sait lire et écrire doit signer (si possible, cette personne devrait être choisie par le participant et devrait n'avoir aucun lien avec l'équipe de recherche). Les participants qui ne savent pas lire ni écrire doivent aussi fournir leur empreinte de pouce.

Bloc de signature les témoins

Ma signature ci-dessous atteste que les informations contenues dans le formulaire de consentement et toute autre information écrite ont été expliquées avec précision au sujet et apparemment comprises par lui, et que le consentement a été donné librement par le sujet.

J'ai été témoin de la lecture exacte du formulaire de consentement à la personne potentiellement participante et elle a eu l'occasion de poser des questions. Je confirme que la personne a donné son consentement éclairé.

**Nom du témoin**	**Signature**	**Date**

Déclaration du chercheur ou de la personne qui recueille le consentement

J'ai lu la fiche d'information au participant potentiel de façon précise et j'ai veillé de mon mieux à ce que la personne comprenne le contenu de l'étude.

Je confirme que la personne a eu l'occasion de poser des questions au sujet de l'étude et que j'ai répondu à ses questions au meilleur de mes connaissances.

Je confirme que la personne n'a pas été forcée à donner son consentement et qu'elle l'a donné de façon libre et volontaire.

Une copie du présent document a été remise à la personne participante.

**Nom de la Personne qui recueille le consentement**	**Signature**	**Date**
