# Supplementary material for: Whole genome sequencing of M. tuberculosis for disease control in high-burden settings: study protocol for a cluster randomized controlled trial evaluating different community-wide intervention strategies in rural Madagascar
Source: Trials. 2024 Oct 25;25:717. doi: 10.1186/s13063-024-08537-4 (PMC11515128; doi:10.1186/s13063-024-08537-4)
Supplement: Supplementary file 3 — Supplementary Material 3. [file 13063_2024_8537_MOESM3_ESM.pdf]

	**TB WGS cRCT Haute Matsiatra**<br>*« Séquençage du Génome Complet de la Tuberculose pour le Contrôle de la Maladie à Madagascar - Un Essai Contrôlé Randomisé en Grappe pour Évaluer Différentes Stratégies d'Intervention à l'Échelle Communautaire »*<br><br>**CAHIER D'OBSERVATION**<br>**CRF00.0_WGScRCT_V3_2023.04.27**

**FR - IDENTIFICATION**<br>*EN - IDENTIFICATION*<br>MG -FAMANTARANA	
**01.FR-Numéro de dossier du participant**<br>*01.EN-Participant file number*<br>01.MG-Laharan'ny dosie ny mpandray anjara	**(DE ou ID ou EP)|__|__|__|__|__|**<br>*(DE or ID or EP)|__|__|__|__|__|*<br>(DE na ID na EP)|__|__|__|__|__|
**02.FR-Numéro d'identifiant correspondant**<br>*02.EN-Corresponding ID number*<br>02.MG-Laharana mifanaraka amin'ny mpandray anjara	◯**dépistage primaire** ◯**domiciliaire**<br>◯**épidémiologique**<br>◯*primary screening* ◯ *domiciliairy*<br>◯*epidemiological*<br>◯fitiliana voalohany ◯ao an-tokatrano<br>◯epidemiolojika
**03.FR - Identité de la personne qui remplit le questionnaire**<br>*03.EN- Identity of the research personnel filling the questionnaire*<br>03.MG- Famantarana ny olona mameno ny andiam-panontaniana	
**04.FR-Identifiant du participant**<br>*04.EN-Partcipant ID number*<br>04.MG-Laharana tokana mpandray anjara<br><br>**FR-Si c'est un participant diagnostiqué dans V1, saisir son PERS V1**<br>*EN- If this is a participant diagnosed in V1, enter their V1 PERS*<br>MG- Raha mpandray anjara voatily tamin'ny V1, ampidiro ny PERS V1	**PERS|__|__|__|__|__|**<br>*PERS|__|__|__|__|__|*<br>PERS|__|__|__|__|__|
**05.FR - Date** de remplissage<br>*05.EN – Filling*<br>05.MG – Daty namenoana ny fisy	**|__|__| / |__|__| / |__|__|__|__| (jj/mm/aaaa)**<br>*|__|__| / |__|__| / |__|__|__|__| (dd/mm/yyyy)*<br>|__|__| / |__|__| / |__|__|__|__| (aa/vv/tttt)
**06.FR-Initial du participant**<br>*06.EN-Participant's initial*<br>06.MG- Fanafohezana ny anarana	**|__|__|__|**<br>*|__|__|__|*<br>|__|__|__|
**07.FR – Date de naissance**<br>*07.EN – Birthdate*<br>07.MG – Daty nahaterahana	**|__|__| / |__|__| / |__|__|__|__| (jj/mm/aaaa)**<br>*|__|__| / |__|__| / |__|__|__|__| (dd/mm/yyyy)*<br>|__|__| / |__|__| / |__|__|__|__| (aa/vv/tt)
**08.FR –Nom du Fokontany**<br>*08.EN – Fokontany* Name<br>08.MG-Anaran' ny Fokontany	

**09.FR – Nom du hameau**<br>*09.EN –Name of Village*<br>09.MG –Anaran'ny Vondrotrano	

Institut Pasteur de Madagascar<br>CRCHUM CENTRE DE RECHERCHE Centre hospitalier de l'Université de Montréal	**TB WGS cRCT Haute Matsiatra**<br>*« Séquençage du Génome Complet de la Tuberculose pour le Contrôle de la Maladie à Madagascar - Un Essai Contrôlé Randomisé en Grappe pour Évaluer Différentes Stratégies d'Intervention à l'Échelle Communautaire »*<br><br>**CAHIER D'OBSERVATION**<br>**CRF1.0_WGScRCT_V4_2022.05.23**

FR – Identification, démographie et profil socioéconomique des Fokontany
EN - Fokontany identification, demographic and socioeconomic informations
MG-Famantarana, toetra ara demografia, sosialy sy toekarena ny Fokontany

**FR – IDENTIFICATION DU FOKONTANY**<br>*EN -FOKONTANY IDENTIFICATION*<br>MG -FAMANTARANA NY FOKONTANY	
**01.FR - Identité de la personne qui remplit le questionnaire**<br>*01.EN- Identity of the research personnel filling the questionnaire*<br>01.MG- Famantarana ny olona mameno ny andiam-panontaniana	
**02.FR - Date**<br>*02.EN - Date*<br>02.MG – Daty	**|__|__| / |__|__| / |__|__|__|__| (jj/mm/aaaa)**<br>*|__|__| / |__|__| / |__|__|__|__| (dd/mm/yyyy)*<br>|__|__| / |__|__| / |__|__|__|__| (aa/vv/tttt)
**03.FR - Numéro d'identification du Fokontany**<br>*03.EN – Fokontany identification number*<br>03.MG – Laharana famantarana ny Fokontany	**|__|__|__|**<br>*|__|__|__|*<br>|__|__|__|
**04.FR - Nom du Fokontany**<br>*04.EN – Name of the Fokontany*<br>04.MG – Anaran'ny Fokontany	
**05.FR – Nombre de village/hameau dans le Fokontany**<br>*05.EN – Number of village in the Fokontany*<br>05.MG – Isan'ny vondrotrano ao amin'ny Fokontany	

**FR – DEMOGRAPHIE ET PROFIL SOCIO-ÉCONOMIQUES FOKONTANY**<br>*EN - FOKONTANY SOCIOECONOMIC AND DEMOGRAPHICINFORMATIONS*<br>MG – MOMBAMOMBA NY ARA-TSOSIALY SY TOEKARENA NY FOKONTANY	
**01.FR – Présence d'un commerce dans le Fokontany**<br>*01.EN – Presence of a traiding shop in the Fokontany*<br>01.MG – Misy magasay (fivarotana) ve ny fokontany ?	◯ **Oui** ◯ **Non**<br>◯ *Yes* ◯ *No*<br>◯ Eny ◯ Tsia
**02.FR – Présence d'un marché dans le Fokontany**<br>*02.EN – Presence of a market in the Fokontany*<br>02.MG – Misy tsena ve ao amin'ny Fokontany ?	◯ **Oui** ◯ **Non**<br>◯ *Yes* ◯ *No*<br>◯ Eny ◯ Tsia
**03.FR – Présence d'une école dans le Fokontany**<br>*03.EN – Presence of school in the Fokontany*<br>03.MG – Misy toeram-pianarana ve ao amin'ny Fokontany ?	◯ **Oui** ◯ **Non**<br>◯ *Yes* ◯ *No*<br>◯ Eny ◯ Tsia

**04.FR – Présence d'un poste de gendarme dans le Fokontany**<br>*04.EN – Presence of a police station in the Fokontany*<br>04.MG – Misy birao ny zandary ve ao amin'ny Fokontany ?	◯ **Oui** ◯ **Non**<br>◯ *Yes* ◯ *No*<br>◯ Eny ◯ Tsia
**05.FR – Présence d'un terrain de foot dans le Fokontany**<br>*05.EN – Presence of football field in the Fokontany*<br>05.MG – Misy kianja fialaovam-baolina kitra ve ao amin'ny Fokontany ?	◯ **Oui** ◯ **Non**<br>◯ *Yes* ◯ *No*<br>◯ Eny ◯ Tsia
**06.FR – Propriétaires de Zébu dans le Fokontany**<br>*06.EN – Zebu owners in the Fokontany*<br>06.MG – Misy manana omby ve ao amin'ny fokontany ?	◯ **Oui** ◯ **Non**<br>◯ *Yes* ◯ *No*<br>◯ Eny ◯ Tsia
**07.FR – Majorité des habitations**<br>*07.EN – Majority of housing buidings*<br>07.MG – Ny akabetsahan'ny trano misy ao amin'ny Fokontany	◯ **Terre** ◯ **Dur**<br>◯ *Mud* ◯ *Hardware*<br>◯Tany ◯ tranovato
**08.FR – Accès au Fokontany**<br>*08.EN – Fokontany acces*<br>08.MG – Ny lalana makany amin'ny Fokontany	◯ **Route carrossable** ◯ **Sentier ou piste**<br>◯ Driveable road ◯ *Track or footpath*<br>◯lalana azo aleha ny fiara ◯lalana azo aleha tongotra
**09.FR – Accès à l'eau dans le Fokontany**<br>*09.EN – Water access in the Fokontany*<br>09.MG – Rano fampiasan'ny olona ao amin'ny Fokontany	◯ **Aqueduc** ◯ **Puit** ◯ **Source naturelle** ◯ **Rivière à aire ouverte**<br>◯ *Running water* ◯ *Well* ◯ *Natural spring* ◯ *Open air river*<br>◯renirano ◯vovo ◯loharano ◯Renirano
**10.FR – Électricité dans le Fokontany**<br>*10.EN – Electricity in the Fokontany*<br>10.MG–Jiro ampesain'ny olona ao amin'ny Fokontany	◯ **Réseau Jirama** ◯ **Panneau solaire** ◯ **Aucun**<br>◯ *Jirama network* ◯ *Solar pannel* ◯ *None*<br>◯Jirama ◯herinaratra avy amin'ny masoandro ◯tsy misy
**11.FR – La majorité des foyers dans le Fokontany possède-t-elle un poste téléviseur ?**<br>*11.EN – Do the majority of households in the Fokontany have television?*<br>11.MG –Mampiasa fahita lavitra ve ny akamaroan'ny olona ao amin'ny Fokontany?	◯ **Oui** ◯ **Non**<br>◯ *Yes* ◯ *No*<br>◯ Eny ◯ Tsia
**12.FR-Est-ce qu'il y a des foyers qui possèdent un récepteur télé satellite (CanalSat ou Startimes)**<br>12.EN- *Do any households have a satellite TV receiver (CanalSat or Startimes)?*<br>*12.MG-Misy olona mampiasa CanalSat na Startimes ve ato amin'ny Fokontany?*	◯ **Oui** ◯ **Non**<br>◯ *Yes* ◯ *No*<br>◯ Eny ◯ Tsia
**13.FR-Existence de couverture de réseau téléphonique**<br>*13.EN-Mobile network coverage*<br>13.MG- Mahazo "reseau" telefonina ve ny Fokontany	◯ **Oui** ◯ **Non**<br>◯ *Yes* ◯ *No*<br>◯ Eny ◯ Tsia
**14. Nombre de population dans le Fokontany**<br>*14.Population number in the Fokontany*<br>14.Isan'ny mponina ao anaty Fokontany	

<table>
<tr><td>Institut Pasteur de Madagascar
CRCHUM CENTRE DE RECHERCHE Centre hospitalier de l'Université de Montréal</td><td>TB WGS cRCT Haute Matsiatra
« Séquençage du Génome Complet de la Tuberculose pour le Contrôle de la Maladie à Madagascar - Un Essai Contrôlé Randomisé en Grappe pour Évaluer Différentes Stratégies d'Intervention à l'Échelle Communautaire »

CAHIER D'OBSERVATION
CRF1.1_WGScRCT_V3_2022.05.14</td></tr>
</table>

FR – Village
EN - Village
MG-Vondrontrano

<table>
<tr><td colspan="2">FR – IDENTIFICATION DU FOKONTANY
EN -FOKONTANY IDENTIFICATION
MG -FAMANTARANA NY FOKONTANY</td></tr>
<tr><td>01.FR - Identité de la personne qui remplit le questionnaire
01.EN- Identity of the research personnel filling the questionnaire
01.MG- Famantarana ny olona mameno ny andiam-panontaniana</td><td></td></tr>
<tr><td>02.FR - Date
02.EN - Date
02.MG – Daty</td><td>|__|__| / |__|__| / |__|__|__|__| (jj/mm/aaaa)
|__|__| / |__|__| / |__|__|__|__| (dd/mm/yyyy)
|__|__| / |__|__| / |__|__|__|__| (aa/vv/tttt)</td></tr>
<tr><td>03.FR - Numéro d'identification du Fokontany
03.EN – Fokontany identification number
03.MG – Laharana famantarana ny Fokontany</td><td>|__|__|__|
|__|__|__|
|__|__|__|</td></tr>
<tr><td>04.FR - Nom du Fokontany
04.EN – Name of the Fokontany
04.MG – Anaran'ny Fokontany</td><td></td></tr>
</table>

<table>
<tr><td colspan="2">FR – POUR CHAQUE VILLAGE
EN – FOR EACH VILLAGE
MG – HO AN'NY VONDROTRANO TSIRAIRAY</td></tr>
<tr><td colspan="2">FR - VILLAGE NUMÉRO 1
EN – VILLAGE NUMBER 1
MG – VONDROTRANO 1</td></tr>
<tr><td>01.FR – Nom du village
01.EN – Village name
01.MG – Anaran'ny Vondrotrano</td><td></td></tr>
<tr><td>02.FR – Population du village
02.EN – Village population
02.MG -Fokon'olona ao anatin'ny vondrotrano</td><td></td></tr>
<tr><td>03.FR – Coordonnées GPS du centre du village
03.EN – Village center GPS coordinates
03.MG – GPS ny ivon'ny vondrotrano</td><td>|__|__|.|__|__|__|__|__| |__|__|.|__|__|__|__|__|
|__|__|.|__|__|__|__|__| |__|__|.|__|__|__|__|__|
|__|__|.|__|__|__|__|__| |__|__|.|__|__|__|__|__|</td></tr>
<tr><td>04.FR – Nom du chef village
04.EN – Village chief name
04.MG – Anaran'ny lehiben'ny vondrotrano</td><td></td></tr>
</table>

**05.FR – Contact du chef village**<br>*05.EN – Contact chief name*<br>05.MG – Laharana an-taroby ny lehibe ny vondrotrano	**032 _ _ _ _ _ _ _**<br>**033 _ _ _ _ _ _ _**<br>**034 _ _ _ _ _ _ _**<br>*032 _ _ _ _ _ _ _*<br>*033 _ _ _ _ _ _ _*<br>*034 _ _ _ _ _ _ _*<br>032 _ _ _ _ _ _ _<br>033 _ _ _ _ _ _ _<br>034 _ _ _ _ _ _ _
**06.FR – Village desservit par agent communautaire**<br>*06.EN – Village covered by a community agent*<br>06.MG – ny vondrotrano sahanin'ny mpanentana ara-pahasalamana<br><br>**FR-SI la réponse est "non", se referer à la question n°8**<br>*EN-If the answer is "no"; refer to the question n°8*<br>MG-Raha toa ka "tsia" ny valiny, tohizana any amin'ny fanontaniana n°8	◯ **Oui** ◯ **Non**<br>◯ *Yes* ◯ *No*<br>◯ Eny ◯ Tsia
**07.FR – Contact de l'agent communautaire**<br>*07.EN – Contact of the community agent*<br>07.MG – laharana an-taroby ny mpanentana ara-pahasalamana	**032 _ _ _ _ _ _ _**<br>**033 _ _ _ _ _ _ _**<br>**034 _ _ _ _ _ _ _**<br>*032 _ _ _ _ _ _ _*<br>*033 _ _ _ _ _ _ _*<br>*034 _ _ _ _ _ _ _*<br>032 _ _ _ _ _ _ _<br>033 _ _ _ _ _ _ _<br>034 _ _ _ _ _ _ _
**08.FR – Distance du village au CSB le plus prêt**<br>*08.EN – Distance from village to closest CSB*<br>08.MG – Fahalaviran'ny CSB akaiky indrindra	**|__|__|__| (distance en kilomètres)**<br>*|__|__|__| (distance in kilometers)* |__|__|__| (halavirana amin'ny kilometatra)
**09.FR – Distance du village au CDT le plus prêt**<br>*09.EN – Distance from village to closest CDT*<br>09.MG – Fahalaviran'ny CDT akaiky indrindra	**|__|__|__| (distance en kilomètres)**<br>*|__|__|__| (distance in kilometers)* |__|__|__| (halavirana amin'ny kilometatra)

TB WGS cRCT Haute Matsiatra
« Séquençage du Génome Complet de la Tuberculose pour le Contrôle de la Maladie à Madagascar - Un Essai Contrôlé Randomisé en Grappe pour Évaluer Différentes Stratégies d'Intervention à l'Échelle Communautaire »

CAHIER D'OBSERVATION
CRF2.0_WGScRCT_V8_2023.05.03

FR – Recherche active de cas
EN – Active case finding
MG- Fitadiavana ireo trangan'aretina raboka

<table>
<tr><td>01.FR-Numéro de dossier du participant
01.EN-Participant file number
01.MG-Laharan'ny dosie ny mpandray anjara</td><td>(DE ou ID ou EP)|__|__|__|__|__|
(DE or ID or EP)|__|__|__|__|__|
(DE na ID na EP)|__|__|__|__|__|</td></tr>
<tr><td>02.FR:Date de remplissage
02.EN: Date of filling
02.MG: daty amenoana ny adiam-panotaniana</td><td>|__|__| / |__|__| / |__|__|__|__| (jj/mm/aaaa)
|__|__| / |__|__| / |__|__|__|__| (dd/mm/yyyy)
|__|__| / |__|__| / |__|__|__|__| (aa/vv/tttt)</td></tr>
<tr><td>03.FR: Numéro d'identifiant correspondant
03.EN: Corresponding ID number
03.MG: Laharana tokana mifanaraka amin'ny mpandray anjara

FR-Si c'est un code DE, renvoi à la question n°7
EN- If it is a DE code, refer to the question n°7
MG- Raha toa ka kaody DE, tohizana avy hatrany any amin'n'ny fanontaniana n°7

FR-Si c'est un code EP, renvoi à la question n°6
EN- If it is an EP code, refer to the question n°6
MG-Raha toa ka kaody EP, tohizana avy hatrany any amin'ny fanontaniana n°6</td><td>◯DE ◯ID ◯EP
◯DE ◯ID ◯EP
◯DE ◯ID ◯EP</td></tr>
<tr><td>04.FR-Ajouter l'Identifiant du cas index
04.EN-Add the ID number of the index case
04.MG-Farito ny kaody famantarana ny tranga fototra</td><td>DE|__|__|__|__|__|
DE|__|__|__|__[__|
DE|__|__|__|__|__|</td></tr>
<tr><td>05.FR-Nature de contact avec le cas index
05.EN-Nature of contact with the index case
05.MG-Farito ny fifandraisana misy amin'ilay tranga fototra</td><td></td></tr>
<tr><td>06.FR: Entrer le numéro de cluster
06.EN: Enter the cluster number
06.MG: Ampidiro ny kaody ny vondrona misy azy</td><td>C|__|__|__|
C|__|__|__|
C|__|__|__|</td></tr>
<tr><td>07.FR – Date de naissance
07.EN – Birthdate
07.MG – Daty nahaterahana</td><td>|__|__| / |__|__| / |__|__|__|__| (jj/mm/aaaa)
|__|__| / |__|__| / |__|__|__|__| (dd/mm/yyyy)
|__|__| / |__|__| / |__|__|__|__| (aa/vv/tt)</td></tr>
<tr><td>08.FR: Age
08.EN: Age
08.MG: Taona</td><td>|__|__| ◯mois ◯année
|__|__| ◯month ◯year
|__|__| ◯volana ◯taona</td></tr>
<tr><td>09.FR-Précison de la date de naissance
09.EN- Precision about birth date
09.MG-Iza no marina amin'ireo</td><td>◯Année ◯Année-mois ◯Année-Mois-Jour
◯Year ◯Year-Month ◯Year-Month-Day
◯Taona ◯Taona-volana ◯Taona-Volana-Andro</td></tr>
</table>

**10.FR-Genre**<br>*10.EN-Gender*<br>10.MG-Fananahana	◯**masculin** ◯**féminin**<br>◯*Male* ◯*Female*<br>◯lahy ◯vavy
**11.FR – Sexe biologique**<br>*11.EN – Biological sexe*<br>11.MG – Fananahana	◯**Masculin** ◯**Féminin**<br>◯*Male* ◯*Female*<br>◯Lahy ◯Vavy
**12.FR- Est ce que vous avez la tuberculose actuellement**<br>*12.EN-Do you have currently tuberculosis?*<br>12.MG- Voan'ny raboka ve ianao izao?<br><br>**FR-Si la réponse est "non", passer à la question n°17**<br>*EN- If the answer is "no", go to the question n°17*<br>MG- Raha "tsia" ny valiny, dia mandeha avy hatrany any amin'ny fanontaniana n°17	◯**Oui** ◯**Non**<br>◯*Yes* ◯*No*<br>◯Eny ◯Tsia
**13.FR-Précisez dans quel CDT vous étiez diagnostiqué**<br>*13.EN-Specify in which CDT you were diagnosed*<br>13.MG-Farito hoe CDT taiza no nanaovana fitiliana	◯**Tambohobe** ◯**Salfa Ivory atsimo** ◯ **Isorana**<br>◯**Alakamisy Itenina** ◯**Mahasoabe**<br>◯**Ambalavao** ◯**Ambohihasoa** ◯**Ikalamavony** ◯**Autre**<br>◯*Tambohobe* ◯*Salfa Ivory atsimo* ◯ *Isorana*<br>◯*Alakamisy Itenina* ◯*Mahasoabe*<br>◯*Ambalavao* ◯*Ambohihasoa* ◯*Ikalamavony* ◯*Other*<br>◯Tambohobe ◯Salfa Ivory atsimo ◯ Isorana<br>◯Alakamisy Itenina ◯Mahasoabe<br>◯Ambalavao ◯Ambohihasoa ◯Ikalamavony ◯Hafa
**14.FR-Préciser le nom du CDT**<br>*14.EN-Specify the name of other CDT*<br>14.MG-Farito ny anaran'ny CDT hafa	
**15.FR-Etes-vous sous traitement TB?**<br>*15.EN-Are you on TB treatment*?<br>15.MG-Manaraka fitsaboana ny raboka ve ianao?<br><br>**FR-Si la réponse est "non", passer à la question n°17**<br>*EN- If the answer is "no", go to the question n°17*<br>MG- Raha "tsia" ny valiny, dia mandeha avy hatrany any amin'ny fanontaniana n°17	◯**Oui** ◯**Non**<br>◯*Yes* ◯*No*<br>◯Eny ◯Tsia
**16.FR-Carte de traitement vu par l'agent de l'étude ?**<br>*16.EN-Treatment card justified by the study officer?*<br>16.MG-Karatra fitsaboana voamarin'ny mpikaroka?<br><br>**FR-Si la réponse est "oui", renvoi au CRF 6.0**<br>*EN- If the answer is "no", refer to the CRF 6.0*<br>MG-Raha toa ka "eny" ny valiny, tohizana any amin'ny CRF 6.0	◯**Oui** ◯**Non**<br>◯*Yes* ◯*No*<br>◯Eny ◯Tsia
**FR - S'il s'agit d'un dépistage domiciliaire ou épidémiologique, vous n'avez pas à colliger les symptômes suivants**<br>*EN - If this is a domiciliary or epidemiological screening, you don't need to question the next symptoms*<br>MG – Raha fitiliana ny ao an-tokatrano na épidemiolojika dia tsy ilaina manontany ireo soritr'aretina etsy ambany	
**FR-Questionnaires des symptômes**<br>EN-Symptoms questionnaires<br>*MG-Andiam-panontaniana momba ny soritr'aretina*	
**17.FR – Toussez-vous depuis au moins 2 semaines?**<br>*17.EN – Are you coughing for at least 2 weeks?*<br>17.MG – Mikohaka efa ho 2 herinandro no mihoatra ve ianao?	◯**Oui** ◯**Non**<br>◯*Yes* ◯*No*<br>◯Eny ◯Tsia

**18.FR - Quand vous toussez, y a-t-il du sang?**<br>*18.EN - When you cough, is there any blood?*<br>18.MG - Rehefa mikohaka ve ianao dia misy rà/lio miaraka aminy?	◯**Oui** ◯**Non**<br>◯*Yes* ◯*No*<br>◯Eny ◯Tsia
**19.FR - Avez-vous eu une fièvre dans les dernières 2 semaines?**<br>*19.EN - Have you had a fever in the last 2 weeks?*<br>19.MG - Efa nisy fotoana voan'ny tazo ve ianao tao anatin'ny 2 herinandro izay?	◯**Oui** ◯**Non**<br>◯*Yes* ◯*No*<br>◯Eny ◯Tsia
**20.FR - Avez-vous perdu du poids dernièrement?**<br>*20.EN – Did you lose weight recently?*<br>20.MG - Mahatsapa fa nien-danja ve ianao?	◯**Oui** ◯**Non**<br>◯*Yes* ◯*No*<br>◯Eny ◯Tsia
**21.FR - Avez-vous des sueurs nocturnes?**<br>*21.EN - Do you have night sweats?*<br>21.MG - Tsemboka ve ianao amin'ny alina?	◯**Oui** ◯**Non**<br>◯*Yes* ◯*No*<br>◯Eny ◯Tsia
**22.FR – Avez-vous déjà été en contact avec un tuberculeux?**<br>*22.EN – Have you been in contact with a tuberculosis patient?*<br>22.MG – Efa nifandray tamin'ny olona voan'ny raboka ve ianao?	◯**Oui** ◯**Non**<br>◯*Yes* ◯*No*<br>◯Eny ◯Tsia
**FR-Participant diagnostiqué par l'étude pendant la V1?**	◯**Oui** ◯**Non**<br>◯*Yes* ◯*No*<br>◯Eny ◯Tsia
**23.FR-Participant éligible pour le dépistage?**<br>*23.EN-Participant eligible for testing?*<br>23.MG-Mahafeno fepetra hanaovana fitiliana ve ny mpandray anjara?<br><br>**FR-Si le participant n'est pas éligible, fin d'investigation**<br>*EN-If the participant is not eligible, end of investigation*<br>MG-Raha tsy mahafeno fepetra ny mpandray anjara, mifarana ny fanadihadiana	◯**Oui** ◯**Non**<br>◯*Yes* ◯*No*<br>◯Eny ◯Tsia
**35.FR-localisation GPS du cas suspect**<br>*35. GPS location of the suspect case*<br>35. GPS ny mpandray anjara misy soritr'aretina	
**FR – Puisqu'au moins un des critères de dépistage est rencontré, le patient doit être testé pour la tuberculose. Si le patient éligible est un enfant de moins de 5 ans ou s'il est incapable de produire un crachat, référer le participant au CDT le plus proche.**<br>*EN – Since at least one screening criteria is present, the patient needs to be tested for TB. If the eligible patient is a child under 5 years old or is unable to produce a sputum sample, refer the participant to the nearest CDT*<br>MG – Rehefa feno ny iray na mihoatra amin'ireo fepetra ny fitiliana raboka, dia tokony hanao fitiliana ny marary. Ho an'ny zaza latsaky ny 5 taona na tsy mahay mandrehoka dia alefa manantona ny CDT akaiky azy izy ireo mba hotiliana.	
**24.FR-Référé au CDT (moins de 5ans)?**<br>*24.EN-Refered to CDT (less than 5years)?*<br>24.MG-Alefa manantona CDT (Latsaky ny 5 taona)?<br><br>**FR-Si la réponse est « non », renvoi à la question n°27**<br>*EN-If the answer is « no », refer to the question n°27*<br>MG-Raha toa ka "tsia" ny valiny; tohizana avy hatrany any amin'ny fanontaniana n°27	◯**Oui** ◯**Non**<br>◯*Yes* ◯*No*<br>◯Eny ◯Tsia
**25.FR-Participant testé au CDT?**<br>*25.EN-Participant tested at CDT?*<br>25.MG-Voatily teny amin'ny CDT ve ny mpandray anjara?	◯**Oui** ◯**Non**<br>◯*Yes* ◯*No*<br>◯Eny ◯Tsia

**26.FR-Résultat du test au CDT**<br>*26.EN-Result of CDT test*<br>26.MG-Valiny fitiliana teny amin'ny CDT<br><br>**FR- Si la réponse est "positif", passer directement au questionnaires CRF 6.0**<br>*EN: If the answer is "posotive", go directly to questionnaires CRF 6.0*<br>MG- Raha "paositifa" ny valiny, tohizo avy hatrany any amin'ny andiam-panontaniana CRF 6.0	◯**Positif** ◯**Négatif**<br>◯*Positive* ◯*Negative*<br>◯Paozitifa ◯Negatifa
**27.FR-1er crachat**<br>*27.EN-1st sputum*<br>27.MG-Rehoka voalohany	◯**oui /** ◯**incapable de cracher /** ◯**refus de cracher / ◯En attente / ◯Crachoir non retourné/ ◯ perdu de vue**<br><br>◯*yes /* ◯*unable to produce sputum/* ◯ *Refusal to produce sputum* ◯*on hold /* ◯*container unreturned /* ◯*lost of sight*<br>◯eny / ◯tsy afaka mandrehoka / ◯ tsy manaiky ny hanome rehoka/ ◯mbola endrasana/ ◯tsy namerina crachoir/ ◯ *tsy hita*
**28.FR-Numéro d'étiquette du 1er crachat**<br>*28.EN-Sticker number of the first sputum*<br>28.MG-Laharan'ny etikety ny rehoka voalohany	
**FR-Confirmation du numéro d'étiquette du 1er crachat**<br>*EN-Confirmation of Sticker number of the first sputum*<br>MG-Fanamarinana ny Laharan'ny etikety ny rehoka voalohany	
**29.FR-Heure et date de collecte de crachats**<br>*29.EN-time and date of sputum collection*<br>29.MG-Ora sy daty nakana ny rehoka	**|__|__| / |__|__| / |__|__| / |__|__| / |__|__|__|__|**<br>**(hh/mm/jj/mm/aaaa)**<br>*|__|__| / |__|__| /|__|__| / |__|__| / |__|__|__|__|*<br>*(hh/mm/dd/mm/yyyy)*<br>|__|__| / |__|__| / |__|__| / |__|__| / |__|__|__|__| (oo/mnmn/aa/vv/tttt)
**30.FR-Heure et date d'envoi du 1er crachat**<br>*30.EN-If yes, time and date the 1st sputum was sent*<br>30.MG-Raha eny, ora sy daty nandefasana ny rehoka voalohany	**|__|__| / |__|__| / |__|__| / |__|__| / |__|__|__|__|**<br>**(hh/mm/jj/mm/aaaa)**<br>*|__|__| / |__|__| /|__|__| / |__|__| / |__|__|__|__|*<br>*(hh/mm/dd/mm/yyyy)*<br>|__|__| / |__|__| / |__|__| / |__|__| / |__|__|__|__| (oo/mnmn/aa/vv/tttt)
**31.FR-2ème crachat**<br>31.EN-2nd sputum<br>31.MG-Rehoka faharoa	◯**oui /** ◯**incapable de cracher /** ◯**refus de cracher / ◯En attente / ◯Crachoir non retourné/ ◯ perdu de vue**<br>◯*yes /* ◯*unable to produce sputum/* ◯ *Refusal to produce sputum* ◯*on hold /* ◯*container unreturned /* ◯*lost of sight*<br>◯eny / ◯tsy afaka mandrehoka / ◯ tsy manaiky ny hanome rehoka/ ◯mbola endrasana/ ◯tsy namerina crachoir/ ◯ *tsy hita*
**32.FR-Numéro d'étiquette du 2ème crachat**<br>*32.EN-Sticker number of the second sputum*<br>32.MG-Laharan'ny etikety ny rehoka faharoa	
**FR-Confirmation du numéro d'étiquette du 2ème crachat**	

EN-Confirmation of Sticker number of the second sputum<br>MG-Fanamarinana ny Laharan'ny etikety ny rehoka faharoa	
**33.FR-Heure et date de collecte de crachats**<br>*33.EN-Time and date of sputum collection*<br>33.MG-Ora sy daty nakana ny rehoka	**|__|__| / |__|__| / |__|__| / |__|__| / |__|__|__|__|**<br>**(hh/mm/jj/mm/aaaa)**<br>*|__|__| / |__|__| /|__|__| / |__|__| / |__|__|__|__|*<br>*(hh/mm/dd/mm/yyyy)*<br>|__|__| / |__|__| / |__|__| / |__|__| / |__|__|__|__| (oo/mnmn/aa/vv/tttt)
**34.FR-Heure et date d'envoi du 2ème crachat**<br>*34.EN-If yes, time and date the 2nd sputum was sent*<br>34.MG-Raha eny, ora sy daty nandefasana ny rehoka faharoa	**|__|__| / |__|__| / |__|__| / |__|__| / |__|__|__|__|**<br>**(hh/mm/jj/mm/aaaa)**<br>*|__|__| / |__|__| /|__|__| / |__|__| / |__|__|__|__|*<br>*(hh/mm/dd/mm/yyyy)*<br>|__|__| / |__|__| / |__|__| / |__|__| / |__|__|__|__| (oo/mnmn/aa/vv/tttt)

Institut Pasteur de Madagascar<br>CRCHUM CENTRE DE RECHERCHE Centre hospitalier de l'Université de Montréal	**TB WGS cRCT Haute Matsiatra**<br>*« Séquençage du Génome Complet de la Tuberculose pour le Contrôle de la Maladie à Madagascar - Un Essai Contrôlé Randomisé en Grappe pour Évaluer Différentes Stratégies d'Intervention à l'Échelle Communautaire »*<br>**CAHIER D'OBSERVATION**<br>**CRF2.1_WGScRCT_V8_1ère partie 2023.07.26**

FR –Informations médicales et profil socioéconomique des participants
EN –medical informations and socioeconomic profile of participants
MG- Mombamomba ara-pahasalamana sy toetra ara-tsosialy sy ara-toekarena

**FR – Ce formulaire doit être complété après l'obtention du consentement libre et éclairé**<br>*EN – This form needs to be completed after obtention of the informed consent*<br>MG – Ireto andiam-panontaniana ireto dia apetraka ao aorian'ny fahazoana ny fanekana an-tsitrapo

**FR –ANTECEDENTS MEDICAUX ET CONTACTS**<br>*EN –PAST MEDICAL HISTORY AND CONTACTS*<br>MG – MOMBAMOBA NY FAHASALAMANA TALOHA SY NY FIFANDRAISANA	
**01.FR - Avez-vous reçu un vaccin contre la Tuberculose (BCG) ?**<br>*01.EN - Have you had an vaccine against tuberculosis (BCG)?*<br>01.MG - Nahavita vaksiny fanefitry ny raboka ve ianao (BCG)?<br><br>**FR-Si la réponse est « non », renvoi à la question n°4**<br>*EN- If the answer is « no », refer to the question n°4*<br>MG-Raha toa ka « tsia » ny valiny, tohizana avy hatrany amin'ny fanontaniana n°4	◯**Oui** ◯ **Non** ◯**Ne sait pas**<br>◯*Yes* ◯ *No* ◯*Do not know*<br>◯Eny ◯ Tsia ◯Tsy hay
**02.FR – Préciser**<br>*02.EN - Specify*<br>02.MG – Farito	◯**Avec preuve (carnet ou carte de vaccination)** ◯**Sans preuve**<br>◯*With proof (vaccinarion card)* ◯*Without proof*<br>◯Misy porofo(karine na karatra vaksiny) ◯Tsy misy porofo
**03.FR - Cicatrice visible ?**<br>*03.EN - Visible scar ?*<br>03.MG - Misy marika ?	◯**Oui** ◯**Non**<br>◯*Yes* ◯*No*<br>◯Eny ◯Tsia
**04.FR - Avez-vous eu dans le passé des signes de tuberculose ? (Toux, perte de poids, fièvre, hémoptysie, sueur nocturne, perte d'appétit)**<br>*04.EN - Have you had signs of tuberculosis in the past ? (cough, weight loss, fever, hemoptysis, night sweats, loss of appetite)*<br>04.MG - Efa nisy fotoana nisy fambara momba ny raboka ve ianao? (kohaka, fienan-danja, tazo, mokoha-drà, tsemboka amin'ny alina, tsy mazoto homana)<br><br>**FR- Si la réponse est « Non », renvoi à la question n°6**<br>*EN- If the answer is « No », refer to the question n°6*<br>MG- Raha toa ka « eny » ny valiny, tohizana vy hatrany amin'ny fanontaniana n°6	◯**Oui** ◯**Non**<br>◯*Yes* ◯*No*<br>◯Eny ◯Tsia

**05.FR - Avez-vous consulté un médecin pour ces symptômes ?**<br>*05.EN – Did you seek medical attention for these symptoms* ?<br>05.MG -Nanantona mpitsabo ve ianao momba izany ?	◯**Oui** ◯**Non**<br>◯*Yes* ◯*No*<br>◯Eny ◯Tsia
**06.FR - Avez-vous dans le passé reçu un diagnostic de tuberculose ?**<br>*06.EN - Have you ever been diagnosed with tuberculosis?*<br>06.MG - Efa avy voamarina ve fa nitondra raboka ianao taloha ?<br><br>**FR-Si la réponse est « non », renvoi à la question n°15**<br>*EN- If the answer is « no », refer to the question n°15*<br>MG- Raha toa ka « tsia »ny valiny, tohizana avy hatrany amin'ny fanontaniana *n°15*	◯**Oui** ◯**Non**<br>◯*Yes* ◯*No*<br>◯Eny ◯Tsia
**07.FR - Avez-vous eu un traitement de la tuberculose ?**<br>*07.EN - Have you recieved a treatment for tuberculosis?*<br>07.MG - Raha eny, nahazo fitsaboana mifandraika amin'izany ve ianao?<br><br>**FR-Si la réponse est «non » ou « ne sait pas », renvoi à la question** n°15<br>*EN- If the answer is « no » or « do not know », refer to the question n°15*<br>MG- Raha toa ka « tsia » na « tsy hay » ny valiny, tohizana avy hatrany amin'ny fanontaniana n°15	◯**Oui** ◯ **Non** ◯**Ne sait pas**<br>◯*Yes* ◯ *No* ◯*Do not know*<br>◯Eny ◯ Tsia ◯Ts hay
**08.FR - Préciser dans quel CDT**<br>*08.EN - Specify in which CDT*<br>08.MG - Farito ny CDT	◯**Tambohobe** ◯**Salfa Ivory atsimo** ◯ **Isorana** ◯**Alakamisy Itenina** ◯**Mahasoabe** ◯**Ambalavao** ◯**Ambohihasoa** ◯**Ikalamavony** ◯**Autre**<br><br>◯*Tambohobe* ◯*Salfa Ivory atsimo* ◯ *Isorana* ◯*Alakamisy Itenina* ◯*Mahasoabe* ◯*Ambalavao* ◯*Ambohihasoa* ◯*Ikalamavony* ◯*Autre*<br><br>◯Tambohobe ◯Salfa Ivory atsimo ◯ Isorana ◯Alakamisy Itenina ◯Mahasoabe ◯Ambalavao ◯Ambohihasoa ◯Ikalamavony ◯Autre
**09.FR-Préciser l'autre CDT**<br>*09.EN- Specify the other CDT*<br>09.MG- Farito ilay CDT hafa	

**10.FR - Année de traitement**<br>*10.EN - Date of treatment*<br>10.MG - Taona ny fitsaboana	**|__|__|__|__| (aaaa)**<br>*|__|__|__|__| (yyyy)*<br>|__|__|__|__| (tttt)
**11.FR - Avez-vous reçu 6 mois ou plus de traitement**<br>*11.EN - Did you receive 6 months or more of treatment*<br>11.MG - Nahavita ny fitsaboana 6 volana na mihoatra ve ianao ?	**◯Oui ◯ Non ◯Ne sait pas**<br>*◯Yes ◯ No ◯Do not know*<br>◯Eny ◯ Tsia ◯Ts hay
**12.FR - Carte traitement confirmée par l'agent d'étude**<br>*12.EN - Treatment card confirmed by the study officer*<br>12.MG - Karatra fanamarihana ny fitsaboana voamarin'ny mpikaroka<br><br>**FR-Si la réponse est « non », renvoi à la question n°15**<br>*EN- If the answer is « no », refer to the question n°15*<br>MG- Raha toa ka « tsia »ny valiny, tohizana avy hatrany amin'ny fanontaniana *n°15*	**◯Oui ◯Non**<br>*◯Yes ◯No*<br>◯Eny ◯Tsia
**13.FR-Issue du traitement**<br>*13.EN-Treatment outcome*<br>13.MG-Vokatry ny fitsaboana	**◯Guéri ◯ Echec ◯Issue inconnu ◯traitement interrompu**<br>*◯Cured ◯ Failed ◯Outcom unknown ◯Treatment interrupted*<br>◯Sitrana ◯Tsy sitrana ◯Tsy hay ◯Tsy tody hatramin'ny farany
**14.FR-En cas d'interruption,préciser la durée de traitement**<br>**14.***EN-In case of interruption,specify the treatment duration*<br>***14.***MG-Rehefa ts nahavita ny fitsaboana hatramin'ny farany, farito ny faharetan'ny Fitsaboana	**|__|__| (mois)**<br>*|__|__| (month)*<br>|__|__| (volana)
**15.FR - Avez-vous dans le passé vécu dans le même logement que quelqu'un atteint de la tuberculose ?**<br>*15.EN - Have you ever lived in the same home with someone infected with tuberculosis?*<br>15.MG - Efa niara-niaina tamin'ny olona voan'ny raboka ve ianao ?<br><br>**FR- Si la réponse est "non" ou « ne sait pas » , renvoi à la question n°17**<br>*EN-If the answer is « No » or "do not know", refer to the question n°17*<br>*MG- raha" tsia" na "tsy hay" ny valiny, tohizana avy hatrany amin'ny fanontaniana n°17*	**◯Oui ◯ Non ◯Ne sait pas**<br>*◯Yes ◯ No ◯Do not know*<br>◯Eny ◯ Tsia ◯Tsy hay
**16.FR - Préciser en quelle année**<br>*16.EN - Specify in which year*<br>16.MG – Farito hoe ovina ? (taona)	**|__|__|__|__| (aaaa)**<br>*|__|__|__|__| (yyyy)*<br>|__|__|__|__| (tttt)

**17.FR - Actuellement, y-a- t-il un sujet atteint de la Tuberculose dans le même logement que vous ?**<br>*17.EN - Is there currently a person with tuberculosis in the same home as you?*<br>17.MG - Misy olona voan'ny raboka ve miara mipetraka aminao amin'izao ?	◯**Oui** ◯ **Non** ◯**Ne sait pas**<br>◯*Yes* ◯ *No* ◯*Do not know*<br>◯Eny ◯ Tsia ◯Ts hay
**18.FR - Y-a- t-il un sujet atteint de la tuberculose dans votre entourage professionnel/ scolaire ?**<br>*18.EN - Is there anyone with tuberculosis in your professional/school environment?*<br>18.MG - Misy olona voan'ny raboka ve manodidina anao any am-piasana na anya m-pianarana ?	◯**Oui** ◯**Non**<br>◯*Yes* ◯*No*<br>◯Eny ◯Tsia
**FR – FACTEURS DE RISQUE DE TUBERCULOSE**<br>*EN – TUBERCULOSIS RISK FACTORS*<br>MG – TATAORATSY MOMBA NY RABOKA	
**19.FR - Est-ce que vous fumez de la cigarette ?**<br>*19.EN - Do you smoke cigarette ?*<br>19.MG - mifoka sigara ve ianao ?<br><br>**FR-Si la réponse est « non », renvoi à la question n°22**<br>*EN- If the answer is « no », refer to the question n°22*<br>MG- Raha toa ka « tsia » ny valiny, tohizana avy hatrany amin'ny fanontaniana n°22	◯**Oui** ◯**Non** ◯**Ne fume plus – je suis sevré**<br>◯*Yes* ◯*No* ◯ *Don't smoke anymore- weaned*<br>◯eny ◯tsia ◯tsy mifoka intsony- efa nijanona
**20.FR - Combien par jour ?**<br>*20.EN - How many per day ?*<br>20.MG - Raha eny, firy isanandro ?	**|__|__|__|**<br>*|__|__|__|*<br>|__|__|__|
**21.FR - Depuis combien d'année ?**<br>*21.EN - Since how many years?*<br>21.MG - Hatramin'ny ovina ?	**|__|__| Année**<br>*|__|__| Year*<br>|__|__| Taona
**22.FR - Consommez-vous du tabac à chiquer (paraky)?**<br>*22.EN - Do you use chewing tobacco ?*<br>22.MG - Minana paraky ve ianao ?	◯**Oui** ◯**Non**<br>◯*Yes* ◯*No*<br>◯Eny ◯Tsia
**23.FR – Êtes-vous infecté par le VIH ?**<br>*23.EN - Are you infected with HIV*<br>23.MG - Mitondra ny tsomik'aretina VIH ?<br><br>**FR- Si la réponse est « non » ou « ne sait pas », renvoi à la question n°29**<br>*EN-If the answer is « no » or « do not know », refer to the question n°29*<br>MG- Raha toa ka « tsia »na « tsy hay » ny valiny, tohizana avy hatrany amin'ny fanontaniana n°29	◯**Oui** ◯ **Non** ◯**Ne sait pas**<br>◯*Yes* ◯ *No* ◯*Do not know*<br>◯Eny ◯ Tsia ◯Ts hay
**24.FR - Stade OMS**<br>*24.EN - If yes, WHO classification*<br>24.MG - Farito ny ambaratonga OMS	◯**1** ◯**2** ◯ **3** ◯**4**<br>◯*1* ◯*2* ◯ *3* ◯*4*<br>◯1 ◯2 ◯ 3 ◯4

**FR-Stade 1: asymptomatique, activité normale**<br>**Stade 2 : symptomatique, activité normale**<br>**Stade 3 : alitement< 50% de la journée au cours de dernier mois**<br>**Stade 4 : alitement> 50 % au cours du dernier mois**<br>*EN-Stage 1: asymptomatic, normal activity*<br>*Stage 2: symptomatic, normal activity*<br>*Stage 3: bed rest < 50% of the day in the last month*<br>*Stage 4: bed rest > 50% in the last month*<br>MG-Dingana 1: tsy misy fambara,matanjaka tsara<br>Dingana 2: misy fambara, matanjaka tsara<br>Dingana 3: am-pandrina <50% ny andro<br>Dingana 4: am-pandrina >50% ny andro	
**25.FR - Prenez-vous un traitement anti-rétroviral ?**<br>*25.EN - Are you taking anti-retroviral treatment ?*<br>25.MG - Manaraka fitsaboana ARV ve ianao?	◯**Oui** ◯ **Non** ◯**Ne sait pas**<br>◯*Yes* ◯ *No* ◯*Do not know*<br>◯Eny ◯ Tsia ◯Ts hay
**26.FR-Taux de CD4 connu ?**<br>**26.EN-CD4 rate ?**<br>**26.MG-Fantatra ve ny tahan'ny CD4?**<br><br>**FR- Si la réponse est « non » ou « ne sait pas », renvoi à la question n°29**<br>*EN-If the answer is « no » or « do not know », refer to the question n°29*<br>MG- Raha toa ka « tsia »na « tsy hay » ny valiny, tohizana avy hatrany amin'ny fanontaniana n°29	◯**Oui** ◯ **Non** ◯**Ne sait pas**<br>◯*Yes* ◯ *No* ◯*Do not know*<br>◯Eny ◯ Tsia ◯Ts hay
**27.FR-Insérer le taux de CD4 le plus récent**<br>*27.EN-Insert the recent CD4 rate*<br>27.MG-Ampidiro ny tahan'ny CD4	**|__|__|__| CD4/mm3**<br>*|__|__|__| CD4/mm3*<br>|__|__|__| CD4/mm3
**28.FR-Date du dernier test CD4**<br>*28.EN-Date of the last CD4 test*<br>*28.MG-Daty ny fizahana CD4 farany*	**|__|__|/|__|__|/|__|__| (jj/mm/aaaa)**<br>*|__|__|/|__|__|/|__|__| (dd/mm/yyyy)*<br>|__|__|/|__|__|/|__|__| (aa/vv/tttt)
**29.FR – Êtes-vous enceinte ?**<br>*29.EN – Are you pregnant ?*<br>29.MG – Mitondra vohoka ve ianao ?	◯**Oui** ◯**Non** ◯**Non applicable**<br>◯*Yes* ◯*No* ◯*Not applicated*<br>◯Eny ◯Tsia ◯Tsy mihatra
**FR – SYMPTÔMES DANS LE DERNIER MOIS**<br>*EN – SYMPTOMS IN THE LAST MONTH*<br>MG –SORITR'ARETINA NANDRITRA NY VOLANA FARANY	
**30.FR - Toux durant plus de 2 semaines**<br>*30.EN - Cough for more than 2 weeks*<br>30.MG – Kohaka mihotra ny 2 herinandro	◯**Oui** ◯**Non**<br>◯*Yes* ◯*No*<br>◯Eny ◯Tsia
**31.FR - Hémoptysie**<br>*31.EN – Coughing blood*<br>31.MG - Kohaka misy rà/lio	◯**Oui** ◯**Non**<br>◯*Yes* ◯*No*<br>◯Eny ◯Tsia

**32.FR - Sentiment de fièvre**<br>*32.EN – Fever feeling*<br>32.MG – Mahatsapa voan'ny tazo	◯**Oui** ◯**Non**<br>◯*Yes* ◯*No*<br>◯Eny ◯Tsia
**33.FR - Fièvre mésurée > 38.0 C**<br>*33.EN - Mesured fever > 38.0 C*<br>33.MG - Rah eny, tazo nohamarinina > 38.0 C	◯**Oui** ◯**Non**<br>◯*Yes* ◯*No*<br>◯Eny ◯Tsia
**34.FR – Perte de poids**<br>*34.EN - Weight loss*<br>34.MG – Fihenan-danja	◯**Oui** ◯ **Non** ◯**Ne sait pas**<br>◯*Yes* ◯ *No* ◯*Do not know*<br>◯Eny ◯ Tsia ◯Tsy hay
**35.FR - Plus de 5 Kg**<br>*35.EN - More than 5 Kg*<br>35.MG - Mihoatra ny 5 kg	◯**Oui** ◯ **Non** ◯**Ne sait pas**<br>◯*Yes* ◯ *No* ◯*Do not know*<br>◯Eny ◯ Tsia ◯Tsy hay
**36.FR - Perte d'appétit**<br>*36.EN - Loss of appetite*<br>36.MG - Malain-komana	◯**Oui** ◯**Non**<br>◯*Yes* ◯*No*<br>◯Eny ◯Tsia
**37.FR - Sueurs nocturnes**<br>*37.EN - Night sweats*<br>37.MG - Tsemboka amin'ny alina	◯**Oui** ◯ **Non** ◯**Ne sait pas**<br>◯*Yes* ◯ *No* ◯*Do not know*<br>◯Eny ◯ Tsia ◯Tsy hay
**38.FR – Dyspnée**<br>*38.EN - Dyspnea*<br>38.MG - Sahirana rehefa miaina	◯**Oui** ◯ **Non** ◯**Ne sait pas**<br>◯*Yes* ◯ *No* ◯*Do not know*<br>◯Eny ◯ Tsia ◯Tsy hay
**39.FR - Douleurs thoraciques**<br>*39.EN - Chest pain*<br>39.MG - Marary tratra	◯**Oui** ◯ **Non** ◯**Ne sait pas**<br>◯*Yes* ◯ *No* ◯*Do not know*<br>◯Eny ◯ Tsia ◯Tsy hay

**FR - CARACTERISTIQUES SOCIO-ECONOMIQUES**<br>*EN – SOCIO-ECONOMIC CHARACTERISTICS*<br>MG – TOETRA ARA-TSOSIALY SY ARA-TOEKARENA	
**40.FR – Scolarité**<br>*40.EN – Education*<br>40.MG – Fianarana	◯**Oui** ◯**Non**<br>◯*Yes* ◯*No*<br>◯Eny ◯Tsia
**41.FR- Niveau**<br>*41.EN-Level*<br>41.MG-Ambaratonga	◯**Primaire terminé**<br>◯**Primaire non terminé**<br>◯**Secondaire terminé ou au-delà**<br>◯**Secondaire non terminé**<br>◯*Primary school completed*<br>◯*Primary school not completed*<br>◯ *High school or more completed*<br>◯*High school not completed*<br>◯Nahavita ny ambaratonga voalohany<br>◯Tsy nahavita ny ambaratonga voalohany<br>◯Nahavita ny ambaratonga faharoa na mihoatra<br>◯Tsy nahavita ny ambaratonga faharoa

**42.FR – Métier**<br>*42.EN - Job*<br>42.MG – Asa	◯**Oui** ◯**Non**<br>◯*Yes* ◯*No*<br>◯Eny ◯Tsia
**43.FR – Préciser l'occupation ou le métier**<br>*43.EN – Specify the occupation or job*<br>*43.MG* – Farito ny asa fivelomana	◯**Employé de bureau (privé ou fonction publique)** ◯**Agriculteur ou fermier**<br>◯**Marchand**<br>◯**Sans emploi**<br>◯**Autres**<br>◯ *Office worker (private or public service)*<br>◯*Farmer*<br>◯*Shopkeeper*<br>◯*Unemployed*<br>◯*Other*<br>◯Mpiasa birao (fanjakana na tsy miankina)<br>◯Mpiompy na mpamboly<br>◯Mpiavarotra<br>◯Tsy miasa<br>◯Hafa
**44.FR – Précisez**<br>*44.EN - Specify*<br>44.MG – Farito	
**45.FR - Nombre de pièces à domicile (en dehors de la cuisine, toilettes et le local pour se laver)**<br>*45.EN - Number of rooms in the house expect the kicthen, toilet and bathroom*<br>45.MG - Firy ny isan'ny efitrano ankoatran'ny lakozia, efitrano fivoahana sy fidiovana	**|__|__|**<br>*|__|__|*<br>|__|__|
**46.FR - Nombre de pièce pour dormir à domicile ?**<br>*46.EN - Number of bedroom in the house*<br>46.MG - isan'ny efitrano fatoriana ao antrano	**|__|__|**<br>*|__|__|*<br>|__|__|
**47.FR - Type de l'habitat**<br>*47.EN - Type of housing*<br>47.MG - Karazan'ny trano ipetrahana	◯**Maison individuelle**<br>◯**Pièces dans une maison avec plusieurs ménages**<br>◯**Autres**<br>◯**Ne sait pas**<br>◯*Individual house*<br>◯*room in a house with several households*<br>◯*Other*<br>◯*Do not know*<br>◯Trano mitokana<br>◯Hefitra itambarana amin'ny olonkafa<br>◯Hafa<br>◯Tsy hay

**48.FR - Caractéristique de logement**<br>**Toit**	◯**Paille** ◯**Tôle** ◯**Tuile** ◯**Ne sait pas**<br>◯**Autres :……………………..**
**Mur**	◯ **Boue** ◯**Bois** ◯**Tôle** ◯**Brique/béton/Pierre**<br>◯**Ne sait pas**
**Sol**	◯ **Terre** ◯**Planche de bois** ◯ **Ciment**<br>◯**Carrelage/ parquet** ◯**Ne sait pas**
48.EN - Housing characteristic<br>*Roof*	◯*Straw* ◯*Sheet metal* ◯*Tile* ◯*Don't know*<br>◯*Other:........................*
Wall	◯*Mud* ◯*Wood* ◯*Sheet metal*<br>◯*Brick/concrete/stone* ◯*Don't know*
Floor	◯*Soil* ◯*Wood board* ◯ *Cement*<br>◯*Tiling/parquet* ◯*Don't know*
48.MG - Momba ny trano ipetrahana<br>Tafo	◯Vondro na bozaka ◯Fanitso ◯Biriky ◯Tsy hay<br>◯Hafa :……………………..
Rindrina	◯ Fotaka ◯Hazo ◯Fanitso<br>◯Birika /vato ◯ Tsy hay
Tany	◯ Tany ◯Gorodona ◯ Sementitra<br>◯Carreau / parquet ◯Tsy hay
**49.FR - Mode principal d'éclairage**<br>*49.EN - Source of lightning*<br>49.MG - Akora manazava ao antrano	◯**Electricité** ◯ **Pétrole** ◯**Panneau solaire**<br>◯**Bougies** ◯**Pas d'éclairage /**◯ **Ne sait pas**<br>◯ **Autres : ………………………………………………..**<br>◯*Electricity* ◯ *Oil* ◯*Solar panel*<br>◯*Candle* ◯*No light* ◯*Do not know*<br>◯*Other : ……………………………………………….*<br>◯Herinaratra ◯ Solitany ◯Hery azo avy amin'ny masoandro<br>◯Labozia ◯Tsy misy ◯Tsy hay<br>◯ Hafa : ……………………………………………….
**50.FR - Mode principal d'approvisionnement en eau**<br>*50.EN – Principal water supply*<br>50.MG - Rano fampiasa	◯**Robinet**<br>◯**Rivière**<br>◯**Puits**<br>◯**Fontaine publique**<br>◯**Source naturelle**<br>◯**Autres** :……………………………………………<br>◯*Tap*<br>◯*River*

	◯*Well*<br>◯*Public fountain*<br>◯*Natural source*<br>◯*Other :…………………………………………….*<br>◯Paompy<br>◯Renirano<br>◯Vovo<br>◯Fatsakana rano<br>◯Loharano<br>◯Hafa :……………………………………………….
**51.FR - Combustible principal de cuisine**<br>*51.EN - Coocking fuel*<br>51.MG - Fitaovana andrahoana	◯**Bois /** ◯ **Charbon de bois/** ◯ **Gaz /**◯**Electricité**<br>◯**Autres : …………………………………….**<br>◯**Ne sait pas**<br>◯*wood/* ◯ *Charcoal/* ◯ *Gases /*◯ *Electricity*<br>◯*Other : …………………………………….*<br>◯*Do not know*<br>◯Kitay/ ◯Saribao/ ◯ Gazy /◯Herinaratra<br>◯Hafa : …………………………………….<br>◯ Tsy hay
**52.FR - Quel type de toilette les membres de votre ménage utilisent-ils habituellement ?**<br>*52.EN - What type of toilet do members of your household usually use?*<br>52MG - manao ahoana ny lava-piringa ampesain'ny mpianakavy ?	◯**Toilettes + chasse d'eau, intérieures**<br>◯**Toilettes + chasse d'eau, extérieures**<br>◯ **Latrines creusées extérieures**<br>◯ **Pas de toilettes / nature**<br>◯ **Autre………………………………………………**<br>◯*Toilet + flush, indoor*<br>◯ *Toilet + flush, outdoor*<br>◯ *Outdoor pit latrine*<br>◯ *No toilet / outside*<br>◯*Other…………………………………………………..*<br>◯Fidiovana misy fisitoman-drano ao anaty trano<br>◯Fidiovana misy fisitoman-drano ivelan'ny trano<br>◯Lavapiringa any antokotany<br>◯Tsy misy/ jangoany<br>◯ Hafa: …………………………………………………
**53.FR - Est-ce que vous avez un local intérieur dédié pour la cuisson**<br>**des repas?**<br>*53.EN - Do you have an indoor dedicated room for cooking?*<br>53.MG - Misy hefitra natokana andrahoana ve anaty antrano ?	◯**Oui** ◯**Non**<br>◯*Yes* ◯*No*<br>◯Eny ◯Tsia
**54.FR - Est-ce que vous avez un local intérieur dédié pour se laver**<br>*54.EN - Do you have an indoor dedicated room for bathing?*	◯**Oui** ◯**Non**<br>◯*Yes* ◯*No*<br>◯Eny ◯Tsia

54.MG – Misy hefitra natokana ho fadrona ve ao anaty trano ?	
**55.FR - Nombre de personnes ayant une activité rémunératrice dans le foyer**<br>*55.EN - Number of income earners in the household*<br>55.MG - Isan'ny olona mampidi-bola ao antrano	**|__|__|** ◯**Ne sait pas**<br>*|__|__|* ◯*Do not know*<br>|__|__| ◯Tsy hay
**56.FR-3ème crachat**<br>*56.EN-3d sputum*<br>56.MG-Rehoka fahatelo	◯**oui /** ◯**En attente /** ◯**Crachoir non retourné/**<br>◯**incapable de cracher/** ◯**refus de cracher**<br><br>◯*yes /* ◯*on hold /* ◯*container unreturned /*<br>◯*unable to produce sputum/* ◯ *Refusal to produce sputum*<br><br>◯eny / ◯mbola endrasana/ ◯tsy namerina crachoir /<br>◯tsy afaka mandrehoka / ◯ tsy manaiky ny hanome rehoka
**57.FR-Numéro d'étiquette du 3ème crachat**<br>*57.EN-Sticker number of the third sputum*<br>57.MG-Laharan'ny etikety ny rehoka fahatelo	
**FR-Confirmation du numéro d'étiquette du 3ème crachat**<br>*EN-Confirmation of Sticker number of the third sputum*<br>MG-Fanamarinana ny Laharan'ny etikety ny rehoka fahatelo	
**58.FR-Heure et date de collecte de crachats**<br>*58.EN-Time and date of sputum collection*<br>58.MG-Ora sy daty nakana ny rehoka	**|__|__| / |__|__| / |__|__| / |__|__| / |__|__|__|__| (hh/mm/jj/mm/aaaa)**<br>*|__|__| / |__|__| /|__|__| / |__|__| / |__|__|__|__| (hh/mm/dd/mm/yyyy)*<br>|__|__| / |__|__| / |__|__| / |__|__| / |__|__|__|__| (oo/mnmn/aa/vv/tttt)
**59.FR-Heure et date d'envoie du 3ème crachat**<br>*59.EN-If yes, time and date the 3rd sputum was sent*<br>59.MG-Raha eny, ora sy daty nandefasana ny rehoka fahatelo	**|__|__| / |__|__| / |__|__| / |__|__| / |__|__|__|__| (hh/mm/jj/mm/aaaa)**<br>*|__|__| / |__|__| /|__|__| / |__|__| / |__|__|__|__| (hh/mm/dd/mm/yyyy)*<br>|__|__| / |__|__| / |__|__| / |__|__| / |__|__|__|__| (oo/mnmn/aa/vv/tttt)

FR –Questionnaires à remplir après 6 mois de traitement anti-tuberculeux
EN - Questionnaires to be completed after 6 months of anti-tuberculosis treatment
MG- Andiam-panontaniana fenoina aorian'ny fitsaboana 6 volana

**FR-Informations sur chaque personne ayant une activité rémunératrice dans le foyer**<br>*EN-Informations about each earner in the household*<br>MG- Mombamomba ny olona mampidi-bola ao an-tokatrano	
**60.FR-Métier**<br>*60.EN- Job/occupations*<br>60.MG-Asa atao	◯**Employé de bureau (privé ou fonction publique)** ◯**Agriculteur ou fermier**<br>◯**Marchand**<br>◯**Sans emploi**<br>◯**Autres**<br>◯ *Office worker (private or public service)*<br>◯*Farmer*<br>◯*Shopkeeper*<br>◯*Unemployed*<br>◯*Other*<br>◯Mpiasa birao (fanjakana na tsy miankina)<br>◯Mpiompy na mpamboly<br>◯Mpiavarotra<br>◯Tsy miasa<br>◯Hafa
**FR-Préciser l'autre**<br>*EN-Specify if other*<br>MG-Farito raha hafa	
**61.FR – Scolarité**<br>*61.EN – Education*<br>61.MG – Fianarana	◯**Oui** ◯**Non**<br>◯*Yes* ◯*No*<br>◯Eny ◯Tsia
**62.FR-Niveau d'étude**<br>*62.EN-Level of education*<br>62.MG-Fari-pahaizana	◯**Primaire terminé**<br>◯**Primaire non terminé**<br>◯**Secondaire terminé ou au-delà**<br>◯**Secondaire non terminé**<br>◯*Primary school completed*<br>◯*Primary school not completed*<br>◯ *High school or more completed*<br>◯*High school not completed*<br>◯Nahavita ny ambaratonga voalohany<br>◯Tsy nahavita ny ambaratonga voalohany<br>◯Nahavita ny ambaratonga faharoa na mihoatra<br>◯Tsy nahavita ny ambaratonga faharoa

**FR-Pour le participant ayant suivi le traitement TB**<br>*EN-For participant who followed TB treatment*<br>MG-Ho an'ny mpandray anjara nanaraka ny fitsaboana TB	
**63.FR-Identifiant du participant**<br>*63.EN-Partcipant ID number*<br>63.MG-Laharana tokana mpandray anjara	**PERS|__|__|__|__|__|**<br>*PERS|__|__|__|__|__|*<br>PERS|__|__|__|__|__|
**64.FR - Numéro de dossier du participant**<br>*64.EN –ID number of participant*<br>64.MG - Laharana famantarana ny mpandray anjara	**◯DE ◯ID ◯EP|__|__|__|__|__|**<br>*◯DE ◯ID ◯EP|__|__|__|__|__|*<br>◯DE ◯ID ◯EP|__|__|__|__|__|
**65.FR - Identité de la personne qui remplit le questionnaire**<br>*65.EN- Identity of the research personnel filling the questionnaire*<br>65.MG- Famantarana ny olona mameno ny andiam-panontaniana	
**66.FR - Date** de remplissage<br>*66.EN – Filling*<br>66.MG – Daty namenoana ny fisy	**|__|__| / |__|__| / |__|__|__|__| (jj/mm/aaaa)**<br>*|__|__| / |__|__| / |__|__|__|__| (dd/mm/yyyy)*<br>|__|__| / |__|__| / |__|__|__|__| (aa/vv/tttt)
**67.FR- Avez-vous reçu un diagnostic de tuberculose ?**<br>*67.EN-have you been diagnosed with TB?*<br>67.MG-Voamarina fa misy raboka ve ianao ?	**◯Oui ◯Non**<br>*◯Yes ◯No*<br>◯Eny ◯Tsia
**68.FR-Date de diagnostic**<br>*68.EN-Diagnostic date*<br>68.MG-Daty nahazoana ny valim-pitiliana	**|__|__| / |__|__| / |__|__|__|__| (jj/mm/aaaa)**<br>*|__|__| / |__|__| / |__|__|__|__| (dd/mm/yyyy)*<br>|__|__| / |__|__| / |__|__|__|__| (aa/vv/tt)
**69.FR - Préciser dans quel CDT**<br>*69.EN - Specify in which CDT*<br>69.MG - Farito ny CDT	**◯Tambohobe ◯Salfa Ivory atsimo ◯ Isorana ◯Alakamisy Itenina ◯Mahasoabe ◯Ambalavao ◯Ambohihasoa ◯Ikalamavony ◯Autre**<br>*◯Tambohobe ◯Salfa Ivory atsimo ◯ Isorana ◯Alakamisy Itenina ◯Mahasoabe ◯Ambalavao ◯Ambohihasoa ◯Ikalamavony ◯Autre*<br>◯Tambohobe ◯Salfa Ivory atsimo ◯ Isorana ◯Alakamisy Itenina ◯Mahasoabe ◯Ambalavao ◯Ambohihasoa ◯Ikalamavony ◯Autre
**70.FR-Préciser l'autre CDT**<br>*70.EN- Specify the other CDT*<br>70.MG- Farito ilay CDT hafa	
**71.FR-Date de début de traitement**<br>*71.EN-treatment start date*<br>71.MG-Daty nanombohana ny fitsaboana	**|__|__| / |__|__| / |__|__|__|__| (jj/mm/aaaa)**<br>*|__|__| / |__|__| / |__|__|__|__| (dd/mm/yyyy)*<br>|__|__| / |__|__| / |__|__|__|__| (aa/vv/tt)

**72.FR-Carte de traitement vérifiée ?**<br>*72.EN-Treatment card checked ?*<br>72.MG-Voamarina ve fa misy karatra fitsaboana ?	**◯Oui ◯Non**<br>*◯Yes ◯No*<br>◯Eny ◯Tsia
**73.FR-Catégorie de traitement**<br>*73.EN-Treatment category*<br>73.MG-Karazan'ny fitsaboana	**◯Nouveaux cas ◯Traitement après échec ◯Perte de vue**<br>*◯New case ◯Treatment after fealure ◯Loss of follow-up*<br>◯Tranga vaovao ◯Fitsaboana tsy nahomby ◯Fitsaboana tsy tody hatramin'ny farany
**74.FR-Durée prévu de traitement**<br>*74.EN-Panned treatment duration*<br>74.MG-Faharetan'ny fitsaboana	**◯6 mois ◯9 mois ◯12 mois**<br>*◯6 months ◯9 months ◯12 months*<br>◯6 volana ◯9 volana ◯12 volana
**75.FR-Traitement terminé ?**<br>*75.EN-Treatment completed ?*<br>75.MG-Nahavita fitsaboana hatrain'ny farany ve ?	**◯Oui ◯Non**<br>*◯Yes ◯No*<br>◯Eny ◯Tsia
**76.FR-Sinon, phase de traitement**<br>*76.EN-If not, Treatment phase*<br>76.MG-raha tsia, dingana misy ny fitsaboana	**◯Phase intensive ◯Phase de poursuite de traitement**<br>*◯Intensive phase ◯Continuation phase*<br>◯ ◯
**77.FR-Durée de traitement**<br>*77.EN-Treatment duration*<br>77.MG-Faharetan'ny fitsaboana	**|__|__|__| mois**<br>*|__|__|__|month* |__|__|__|volana
**78.FR-Type de centre de santé qui dispense le traitement**<br>*78.EN- Type of health centre providing the treatment*<br>78.MG-Karazana toeram-pitsaboana anaovana ny fitsaboana	**◯Centre de santé de base ◯Centre hospitalier de District ◯Centre hospitalier universitaire**<br>*◯Primary healthcare center ◯District hospital ◯University hospital center*<br>◯CSB ◯CHRD ◯CHU
**79.FR-Mode de traitement**<br>*79.EN-Modality of treatment*<br>79.MG-Fomba fitsaboana	**◯traitement directement observé ◯Traitement non surveillé**<br>*◯Directly observed therapy ◯Self administration*<br>◯Fitsaboana arahi-maso ◯Fitsaboana tsy voahara-maso

**FR-HOPITALISATION**<br>*EN-HOSPITALIZATION*<br>MG-FIDIRANA HOPITALY	
**80.FR-Avez-vous été hospitalisé ?**<br>*80.EN-Have you been hospitalized?*<br>80.MG-Niditra hopitaly ve ianao?	**◯Oui ◯Non**<br>*◯Yes ◯No*<br>◯Eny ◯Tsia
**81.FR-Si oui, combien de jours ?**<br>*81.If yes, how many days?*<br>81. Raha eny, firy andro?	**|__|__|__|**<br>*|__|__|__|* |__|__|__|
**82.FR-Frais d'hébergement pendant l'hospitalisation ?**<br>*82.EN-Accommodation fees during hospitalization?*	

82.MG-Vola lany amin'ny fipetrahana eny amin'ny hopitaly nandritra ny fitsaboana?	
**83.FR-Frais de médicaments durant l'hospitalisation**<br>*83.EN-Drugs fees during hospitalization*<br>83.MG-Vola lany tamin'ny vidim-panafody nandritra ny fitsaboana	
**84.FR-Frais de repas durant l'hospitalisation**<br>*84.EN-Foods fees during hospitalization*<br>84.MG-Vola lany tamin'ny sakafo nandritra ny fitsaboana	
**85.FR-Coût des analyses**<br>*85.EN-Cost of differents tests*<br>85.MG-Vidin'ny fitiliana natao	
**86.FR-Coût des déplacements durant l'hospitalisation**<br>*86.EN-Cost of transportation during hospitalization*<br>86.MG-Vola lany tamin'ny fivezivezena nandritra ny fitsaboana teny amin'ny hopitaly	
**87.FR-Autres dépenses durant l'hospitalisation**<br>*87.EN-Other expenses during hospitalization*<br>87.MG-Fandaniana hafa nandritra ny fipetrahana amin'ny hopitaly	◯**Oui** ◯**Non**<br>◯*Yes* ◯*No*<br>◯Eny ◯Tsia
**88.FR-Si oui, précisez**<br>*88.EN-If yes, specify*<br>88.MG-Raha eny, farito	
**89.FR-Si oui, précisez le coût**<br>*89.EN-If yes, specify cost*<br>89.MG-Raha eny, farito hoe oatrinona	
**90.FR-Combien de temps avez-perdu durant l'hospitalisation par rapport à votre occupation ?**<br>*90.EN- How much time was lost during the hospitalization in relation to your occupation?*<br>90.MG-Fotoana very tsy niasana nandritra ny fitsaboana teny amin'ny hopitaly?	**|__|__|** ◯**Jours** ◯**Mois** ◯**Non applicable**<br>*|__|__|* ◯*Days* ◯*Months* ◯*No applicable*<br>|__|__| ◯Andro ◯Volana ◯Tsy mihatra
**91.FR-Combien de personnes se sont occupées de vous pendant votre hospitalisation**<br>*91.EN-How many people were involved in looking after you while you were hospitalized?*<br>91.MG-Olona firy no nikarakara anao teny amin'ny hopitaly	**|__|__|**<br>*|__|__|*<br>|__|__|
**92.FR-Ces personnes ont-elles une activité rémunératrice pour le foyer ?**<br>*92.FR-Do these people are an income-earners in the household?*	◯**Oui** ◯**Non**<br>◯*Yes* ◯*No*<br>◯Eny ◯Tsia

92.MG-Mpapidi-bola ao an-tokatrano ve ireo olona ireo?	
**93.FR-Temps perdus par les autres membres du foyer ayant une activité rémunératrice durant votre hospitalisation**<br>*93.EN- Time lost by other income earners in the household during your hospitalization*<br>93.MG-Fotoana very ho an'ny olona mampidi-bola ao antrano nandritra ny fitsaboana teny amin'ny hopitaly	**|__|__| ◯Jours ◯Mois ◯Non applicable**<br>*|__|__| ◯Days ◯Months ◯No applicable*<br>|__|__| ◯Andro ◯Volana ◯Tsy mihatra

**FR-TRAITEMENT EXTRAHOSPITALIER**<br>*EN-OUT-PATIENT TREATMENT*<br>MG-FITSABOANA IVELAN'NY HOPITALY	
**94.FR-Frais d'hébergement si applicable**<br>*94.EN-accommodation costs if applicable*<br>94.MG-Vola lany amin'ny hofantrano raha nisy	**________________ ◯Non applicable**<br>*________________ ◯No appicable*<br>________________ ◯Tsy nisy
**95.FR-Coût pour les autres médicaments durant le traitement**<br>*95.EN-Other drugs fees during treatment*<br>95.MG-Vola lany tamin'ny vidim-panafody hafa nandritra ny fitsaboana	**________________ ◯Non applicable**<br>*________________ ◯No appicable*<br>________________ ◯Tsy nisy
**96.FR-Frais de repas durant le traitement**<br>*96.EN-Foods fees during treatment*<br>96.MG-Vola lany tamin'ny sakafo nandritra ny fitsaboana	**________________ ◯Non applicable**<br>*________________ ◯No appicable*<br>________________ ◯Tsy nisy
**97.FR-Coût des analyses**<br>*97.EN-Cost of differents tests*<br>97.MG-Vidin'ny fitiliana natao	**________________ ◯Non applicable**<br>*________________ ◯No appicable*<br>________________ ◯Tsy nisy
**98.FR-Coût des déplacements durant le traitement**<br>*98.EN-Cost of transportation during treatment*<br>98.MG-Vola lany tamin'ny fivezivezena nandritra ny fitsaboana	**________________ ◯Non applicable**<br>*________________ ◯No appicable*<br>________________ ◯Tsy nisy
**99.FR-Autres dépenses durant le traitement**<br>*99.EN-Other expenses during the treatment*<br>99.MG-Fandaniana hafa nandritra ny fitsaboana	**◯Oui ◯Non**<br>*◯Yes ◯No*<br>◯Eny ◯Tsia
**100.FR-Si oui, précisez**<br>*100.EN-If yes, specify*<br>100.MG-Raha eny, farito	
**101.FR-Si oui, précisez le coût**<br>*101.EN-If yes, specify cost*<br>101.MG-Raha eny, farito hoe oatrinona	

**102.FR-Devez-vous interrompre votre activité pour aller récupérer les médicaments à l'hôpital ?**<br>*102.EN- Do you have to interrupt your occupation to pick up the drugs to the hospital?*<br>102.MG-Mila manapaka ny asanao ve ianao rehefa haka ny fanafody eny amin'ny hopitaly?	◯**Oui** ◯**Non**<br>◯*Yes* ◯*No*<br>◯Eny ◯Tsia
**103.FR-Combien de temps par jour perdez-vous pour récupérer les médicaments à l'hôpital ?**<br>*103.EN-How much time per day do you spend to pick up drugs from the hospital?*<br>103.MG-Adiny firy no laninao hakana ny fanafody eny am toeram-pitsaboana?	**|__|__|__| heures**<br>*|__|__|__|hours*<br>|__|__|__|ora
**104.FR-Avez-vous besoin de quelqu'un pour vous remplacer pendant votre absence ?**<br>*104.FR-Do you need someone to replace you while you are absent?*<br>*104.MG-Mila olona ve ianao misolo anao rehefa tsy eo ianao?*	◯**Oui** ◯**Non**<br>◯*Yes* ◯*No*<br>◯Eny ◯Tsia
**105.FR-Si oui, combien cela vous coûte durant le traitement ?**<br>*105.FR-If so, how much does it cost during treatment?*<br>105.MG-Raha eny, oatrinona no laninao amin'izany mandritra ny fitsaboana?	________________ ◯**Non applicable**<br>________________ ◯*No applicable*<br>________________ ◯Tsy nisy

**FR-CONTROLE**<br>*EN-CONTROL*<br>MG-FANARAHAMASO	
**106.FR-Combien de fois faites-vous des contrôles à l'hôpital ?**<br>*106.EN-How often do you have control at the hospital?*<br>106.MG-Impiry ianao no mandeha manao fanaraha-maso ny aretina?	**|__|__|**<br>*|__|__|*<br>|__|__|
**107.FR-Coût des analyses**<br>*107.EN-Cost of differents tests*<br>107.MG-Vidin'ny fitiliana natao	________________ ◯**Non applicable**<br>________________ ◯*No applicable*<br>________________ ◯Tsy nisy
**108.FR-Coût des déplacements pour le contrôle**<br>*108.EN-travel costs for the control*<br>108.MG-Vola lany tamin'ny fivezivezena amin'ny fanarahamaso ny aretine	________________ ◯**Non applicable**<br>________________ ◯*No applicable*<br>________________ ◯Tsy nisy
**109.FR-Autres dépenses pour le contrôle**<br>*109.EN-Other expenses for the control*<br>109.MG-Fandaniana hafa nandritra ny fanarahamaso	◯**Oui** ◯**Non**<br>◯*Yes* ◯*No*<br>◯Eny ◯Tsia

**110.FR-Si oui, précisez**<br>*110.EN-If yes, specify*<br>110.MG-Raha eny, farito	
**111.FR-Si oui, précisez le coût**<br>*111.EN-If yes, specify cost*<br>111.MG-Raha eny, farito hoe oatrinona	
**112.FR-Devez-vous interrompre votre activité pour votre contrôle à l'hôpital ?**<br>*112.EN- Do you have to interrupt your occupation for your control to the hospital?*<br>112.MG-Mila manapaka ny asanao ve ianao rehefa hanao ny fanarahamaso ny aretina eny amin'ny hopitaly?	◯**Oui** ◯**Non**<br>◯*Yes* ◯*No*<br>◯Eny ◯Tsia
**113.FR-Combien de temps par jour perdez-vous pour votre contrôle à l'hôpital ?**<br>*113.EN-How much time per day do you spend for your control the CDT/CSB?*<br>113.MG-Adiny firy no laninao ho an'ny fanarahamaso ny aretina eny am toeram-pitsaboana?	**|__|__|__| heures**<br>*|__|__|__|hours*<br>|__|__|__|ora
**114.FR-Avez-vous besoin de quelqu'un pour vous remplacer pendant votre absence ?**<br>*114.FR-Do you need someone to replace you while you are absent?*<br>*114.MG-Mila olona ve ianao misolo anao rehefa tsy eo ianao?*	◯**Oui** ◯**Non**<br>◯*Yes* ◯*No*<br>◯Eny ◯Tsia
**115.FR-Si oui, combien cela vous coûte durant le traitement ?**<br>*115.FR-If so, how much does it cost during treatment?*<br>115.MG-Raha eny, oatrinona no laninao amin'izany mandritra ny fitsaboana?	**_________________** ◯**Non applicable**<br>_________________ ◯*No applicable*<br>_________________ ◯Tsy nisy

**FR-IMPACTS SOCIO-ECONOMIQUES DE LA MALADIE**<br>*EN-SOCIO-ECONOMIC IMPACTS OF THE DISEASE*<br>MG-FIANTRAKANY ARA TOEKARENA SY SOSIALY NATERAKY NY ARETINA	
**116.FR-Impact financier**<br>*116.EN-Financial impact*<br>116.MG-Fiantraikany ara-bola	◯**Pas d'impact** ◯**Faible impact** ◯**impact modéré** ◯**important impact**<br>◯*No impact* ◯*little impact* ◯*Moderate impact* ◯*serious impact*<br>◯Tsisy fiantraikany ◯Misy fiantraikany kely ◯Eo ho eo ny fiantraikany ◯Misy fiantraikany be
**117.FR-Mécanisme d'adaptation pendant le traitement**<br>*117.EN-Adaptation mechanisms during treatment*	◯**Prêt** ◯**Vente des biens** ◯ **demande d'assistance financière auprès des associations ou organisation** ◯**Autres** ◯**Pas de changement**

117.MG-Fomba fiatrehana ny fitsaboana	◯*Loan* ◯*Sell of assets* ◯*application for financial support from associations or organisations* ◯*Other* ◯*No adaptation*<br>◯Miandram-bola ◯Mamarotra fananana ◯Fangatahana fanampiana avy amin'ireo fikambanana malala-tanana ◯Hafa ◯Tsy misy fiovana
**FR-SI autre, précisez**<br>*EN-If other, specify*<br>MG-Raha hafa, farito	
**118.FR-Impact social**<br>*118.EN-Social impact*<br>118.MG-Fiantraikany ara-tsosialy	◯**Stigmatisation** ◯**Perte d'emploi** ◯**Arrêt ou interruption de la scolarisation** ◯**Séparation/divorce** ◯**Pas d'impact** ◯**Autres**<br>◯*Social exclusion* ◯*Job loss* ◯ *Stopor interrupted schooling* ◯*Séparation/divorce* ◯*No impact* ◯*Other*<br>◯Fanilikilihana ara-piaramonina ◯Very asa ◯ Tapaka na nijanona fianarana ◯Nisara-bady ◯Tsisy fiantraikany ◯Hafa
**119.FR-Impact économique**<br>*119.EN-Economic impact*<br>119.MG-Fiantraikany ara toekarena	◯**Inchangé** ◯**Appauvrissment**<br>◯*Unchanged* ◯*Poorer*<br>◯Tsisy fiovana ◯Nihanahantra

Institut Pasteur de Madagascar<br>CRCHUM CENTRE DE RECHERCHE Centre hospitalier de l'Université de Montréal	**TB WGS cRCT Haute Matsiatra**<br>*« Séquençage du Génome Complet de la Tuberculose pour le Contrôle de la Maladie à Madagascar - Un Essai Contrôlé Randomisé en Grappe pour Évaluer Différentes Stratégies d'Intervention à l'Échelle Communautaire »*<br><br>**CAHIER D'OBSERVATION**<br>**CRF2.2_WGScRCT_V1_2023.05.03**

FR – Informations sur les tuberculeux diagnostiqués dans l'étude

EN- Information on tuberculosis patients diagnosed in the study

MG-Mombamomba ny mpandray anjara voatily mitondra ny raboka ato anaty fanadihadiahana

**FR-Information sur le tuberculeux V1**<br>*EN- Information on tuberculosis patients diagnosed in V1*<br>MG--Mombamomba ny mpandray anjara voatily mitondra ny raboka nandritra ny V1	
**01.FR-Participant diagnostiqué par l'étude pendant la V1?**<br>*01.EN- tuberculosis patient diagnosed in V1 ?*<br>*01.*MG-Mpandray anjara voatily mitondra raboka tao amin'ny V1?<br>**FR-Si la réponse est « non », renvoi au Q°06**<br>*EN- If the answer is "no", refer to Q°06*<br>MG-Raha « Tsia » ny valiny, tohizana amin'ny fanontaniana n°06	**Oui** ◯**Non**<br>◯*Yes* ◯*No*<br>◯Eny ◯Tsia
**02.FR-Est ce que vous avez suivi un traitement?**<br>*02.EN--Have you taken any treatment?*<br>02.MG- Nanaraka fitsaboana mifandraika amin'ny raboka ve ianao?<br><br>**FR-Si la réponse est « non », renvoi au CRF 6.0**<br>*EN- If the answer is "no", refer to CRF 6.0*<br>MG-Raha « Tsia » ny valiny, tohizana amin'ny fanontaniana CRF 6.0	◯**Oui** ◯**Non**<br>◯*Yes* ◯*No*<br>◯Eny ◯Tsia
**03.FR- Avez-vous terminé le traitement?**<br>*03.EN- Have you completed the treatment?*<br>*03.*MG-Nahavita fitsaboana hatramin'ny farany ve ianao?<br><br>**FR-Si la réponse est « non », renvoi au Q°05**<br>*EN- If the answer is "no", refer to Q°05*<br>MG-Raha « Tsia » ny valiny, tohizana amin'ny fanontaniana n°05	◯**Oui** ◯**Non**<br>◯*Yes* ◯*No*<br>◯Eny ◯Tsia
**04.FR-Carte/carnet justifiant la guérison vérifiée par l'agent de l'étude**<br>*04.EN- Treatment card proving recovery justified by the study officer?*<br>04.MG- Karatra nahavitana fitsaboana voamarin'ny mpikaroka?	◯**Oui** ◯**Non**<br>◯*Yes* ◯*No*<br>◯Eny ◯Tsia
**05.FR-Carte de suivi de traitement justifiée par l'agent de l'étude**<br>*05.EN--Treatment card justified by the study*	◯**Oui** ◯**Non**<br>◯*Yes* ◯*No*<br>◯Eny ◯Tsia

officer?<br>*05.*MG- Karatra fitsaboana voamarin'ny mpikaroka?	
**FR-Comme le résultat de dépistage est positif, procéder à l'investigation intradomiciliaire**<br>*EN-* As *the screening result is positive, proceed with the intradomiciliary investigation.*<br>MG-Rehefa pozitiva ny valin'ny fitiliana, tohizana amin'ny fanadihadiana ny olona ao antokatrano	

**FR-Information sur le tuberculeux V2**<br>*EN- Information on tuberculosis patients diagnosed in V2*<br>MG--Mombamomba ny mpandray anjara voatily mitondra ny raboka nandritra ny V2	
**06.FR-Est-ce que c'est un participant recensé lors de la V1 ?**<br>*06.EN-Is this a participant surveyed during V1?*<br>06.MG-Efa voahadihady tany amin'ny V1 ve ny mpandray anjara?<br><br>**FR-Si la réponse est « non », renvoi au CRF 6.0**<br>*EN- If the answer is "no", refer to CRF 6.0*<br>MG-Raha « Tsia » ny valiny, tohizana amin'ny fanontaniana CRF 6.0	◯**Oui** ◯**Non**<br>◯*Yes* ◯*No*<br>◯Eny ◯Tsia
**07.FR-Saisir son identifiant unique V1**<br>*07.EN- Enter the V1 ID*<br>07.MG-Ampidiro ny laharana famantarana manokana tany amin'ny V1	**PERS|__|__|__|__|__|**

TB WGS cRCT Haute Matsiatra
« Séquençage du Génome Complet de la Tuberculose pour le Contrôle de la Maladie à Madagascar - Un Essai Contrôlé Randomisé en Grappe pour Évaluer Différentes Stratégies d'Intervention à l'Échelle Communautaire »

CAHIER D'OBSERVATION
CRF3.0_WGScRCT_V6_2023.03.22

FR- Résultats de laboratoire
EN- Laboratory results
MG-Vokatry ny labotatoara

<table>
<tr><th colspan="2">FR- EXAMEN MICROSCOPIQUE CDT FIANARANTSOA
EN- CDT FIANARANTSOA MICROSCOPY
MG- FANADINANA MIKROSKOPIKA</th></tr>
<tr><td>01.FR- Crachat 1er jour
01.EN- 1st day suptum
01.MG- Rehoka andro voalohany</td><td>◯ Oui ◯ Non
◯ Yes ◯ No
◯ Eny ◯ Tsia</td></tr>
<tr><td>02.FR- Date de prélèvement J1
02.EN- Sampling date J1
02.MG- Daty nanaovana ny santionana J1</td><td>|__|__|/|__|__|/|__|__|__|__| (jj/mm/aaaa)
|__|__|/|__|__|/|__|__|__|__| (dd/mm/yyyy)
|__|__|/|__|__|/|__|__|__|__| (aa/vv/tttt)</td></tr>
<tr><td>03.FR- Heure du prélèvement J1
03.EN- Sampling hour J1
03.MG- Ora nanaovana ny santionana J1</td><td>|__|__|/|__|__| (hh:mm)
|__|__|/|__|__| (hh:mm)
|__|__|/|__|__| (oo:mm)</td></tr>
<tr><td>04.FR- Date de réception CDT Fianarantsoa J1
04.EN- Date of receipt By CDT Fianarantsoa J1
04.MG- Daty nandraisan'ny CDT Fianarantsoa J1</td><td>|__|__|/|__|__|/|__|__|__|__| (jj/mm/aaaa)
|__|__|/|__|__|/|__|__|__|__| (dd/mm/yyyy)
|__|__|/|__|__|/|__|__|__|__| (aa/vv/tttt)</td></tr>
<tr><td>05.FR- Observation qualitatif J1
05.EN- Quality observation J1
05.MG- Fahitana ny kalitao J1</td><td>◯ Salivaire ◯ Purulent ◯ Muqueux ◯ Mucopurulent ◯ Hémoptoique
◯ Salivary ◯ Purulent ◯ Mucosal ◯ Mucopurulent ◯ Hemoptoic
◯ Rora ◯ Nana ◯ Madity ◯ Madity sady misy nana ◯ Misy rà</td></tr>
<tr><td>06.FR- Volume J1
06.EN- Volume J1
06.MG- Fatra J1</td><td>|__|__| (mL)
|__|__| (mL)
|__|__| (mL)</td></tr>
<tr><td>07.FR- Date de manipulation J1
07.EN-Handling date J1
07.MG- Daty nanaovana azy J1</td><td>|__|__|/|__|__|/|__|__|__|__| (jj/mm/aaaa)
|__|__|/|__|__|/|__|__|__|__| (dd/mm/yyyy)
|__|__|/|__|__|/|__|__|__|__| (aa/vv/tttt)</td></tr>
<tr><td>08.FR-Examen direct Ziehl-Neelsen J1
08.EN- Ziehl-Neelsen Microscopy J1
08.MG- Ziehl-Neelsen mikroskopia J1</td><td>◯ Négatif ◯ Faible positif ◯ 1+ ◯ 2+ ◯ 3+ ◯ Non Fait
◯ Negative ◯ Low positive ◯ 1+ ◯ 2+ ◯ 3+ ◯ Not done
◯ Tsisy ◯ Misy kely ◯ 1+ ◯ 2+ ◯ 3+ ◯ Tsy natao</td></tr>
<tr><td>FR-Si ziehl-Neelsen " Faible positif ", précisez le nombre de BAAR trouvés J1
EN- If ziehl-Neelsen 'Low positive', specify the number of BAARs found in D1
MG-Raha toa ka ''Misy kely'' dia omeo ny isa hita J1</td><td></td></tr>
<tr><td>09.FR- Date de rendu résultat J1
09.EN- Date of results J1
09.MG- Daty nanolorana ny vokatra J1</td><td>|__|__|/|__|__|/|__|__|__|__| (jj/mm/aaaa)
|__|__|/|__|__|/|__|__|__|__| (dd/mm/yyyy)
|__|__|/|__|__|/|__|__|__|__| (aa/vv/tttt)</td></tr>
</table>

**10.FR- Crachat 2ème jour**<br>*10.EN- 2nd suptum*<br>10.MG- Rehoka faharoa	◯ Oui ◯ Non<br>◯ *Yes* ◯ *No*<br>◯ Eny ◯ Tsia
**11.FR- Date de prélèvement J2**<br>*11.EN- Sampling date J2*<br>11.MG- Daty nanaovana ny santionana J2	**|__|__|/|__|__|/|__|__|__|__| (jj/mm/aaaa)**<br>*|__|__|/|__|__|/|__|__|__|__| (dd/mm/yyyy)*<br>|__|__|/|__|__|/|__|__|__|__| (aa/vv/tttt)
**12.FR- Heure du prélèvement J2**<br>*12.EN- Sampling hour J2*<br>12.MG- Ora nanaovana ny santionana J2	**|__|__|/|__|__| (hh:mm)**<br>*|__|__|/|__|__| (hh:mm)*<br>|__|__|/|__|__| (oo:mm)
**13.FR- Date de réception CDT Fianarantsoa**<br>*13.EN- Date of receipt By CDT Fianarantsoa*<br>13.MG- Daty nandraisan'ny CDT Fianarantsoa	**|__|__|/|__|__|/|__|__|__|__| (jj/mm/aaaa)**<br>*|__|__|/|__|__|/|__|__|__|__| (dd/mm/yyyy)*<br>|__|__|/|__|__|/|__|__|__|__| (aa/vv/tttt)
**14.FR- Observation qualitatif J2**<br>*14.EN- Quality observation J2*<br>14.MG- Fahitana ny kalitao J2	◯ **Salivaire** ◯ **Purulent** ◯ **Muqueux** ◯ **Mucopurulent** ◯ **Hémoptoique**<br>◯ *Salivary* ◯ *Purulent* ◯ *Mucosal* ◯ *Mucopurulent* ◯ *Hemoptoic*<br>◯ Rora ◯ Nana ◯ Madity ◯ Madity sady misy nana ◯ Misy rà
**15.FR- Volume J2**<br>*15.EN- Volume J2*<br>15.MG- Fatra J2	**|__|__| (mL)**<br>*|__|__| (mL)*<br>|__|__| (mL)
**16.FR- Date de manipulation J2**<br>*16.EN-Handling date J2*<br>16.MG- Daty nanaovana azy J2	**|__|__|/|__|__|/|__|__|__|__| (jj/mm/aaaa)**<br>*|__|__|/|__|__|/|__|__|__|__| (dd/mm/yyyy)*<br>|__|__|/|__|__|/|__|__|__|__| (aa/vv/tttt)
**17.FR-Examen direct Ziehl-Neelsen J2**<br>*17.EN- Ziehl-Neelsen Microscopy J2*<br>17.MG- Ziehl-Neelsen mikroskopia J2	◯ **Négatif** ◯ **Faible positif** ◯ **1+** ◯ **2+** ◯ **3+** ◯ **Non Fait**<br>◯ *Negative* ◯ *Low positive* ◯ *1+* ◯ *2+* ◯ *3+* ◯ *Not done*<br>◯ Tsisy ◯ Misy kely ◯ 1+ ◯ 2+ ◯ 3+ ◯ Tsy natao
**FR-Si ziehl-Neelsen " Faible positif ", précisez le nombre de BAAR trouvés J2**<br>*EN- If ziehl-Neelsen 'Low positive', specify the number of BAARs found in D2*<br>MG-Raha toa ka ''Misy kely'' dia omeo ny isa hita J2	
**18.FR- Date de rendu résultat J2**<br>*18.EN- Date of results J2*<br>18.MG- Daty nanolorana ny vokatra J2	**|__|__|/|__|__|/|__|__|__|__| (jj/mm/aaaa)**<br>*|__|__|/|__|__|/|__|__|__|__| (dd/mm/yyyy)*<br>|__|__|/|__|__|/|__|__|__|__| (aa/vv/tttt)
**FR- TEST XPERT MTB/RIF ULTRA**<br>*EN- XPERT MTB/RIF ULTRA TEST*<br>MG-FITILIANA XPERT MTB/RIF ULTRA	
**FR- Si 2 crachats sont disponibles, effectuer l'analyse Xpert sur le crachat le plus purulent / sanguin**<br>*EN- If 2 suptum samples are avalaibl, perform Xpert on the most purulent/hemoptoic sample*<br>MG - Raha toa ka anananana ny rehoka roa dia atao amin'izay be nana sy misy rà ny fitiliana	
**19.FR-Numéro du crachat**<br>*19.EN-Suptum Number*<br>19.MG- Laharan'ny rehoka	◯ **1er** ◯ **2ème**<br>◯ *1st* ◯ *2nd*<br>◯ Voalohany ◯ Faharoa
**20.FR-Test GeneXpert (Test #1)**<br>*20.EN- GeneXpert test (Test #1)*<br>20.MG- Fitiliana GeneXpert (Test #1)	◯ **Oui** ◯ **Non**<br>◯ Yes ◯ No<br>◯ Eny ◯ Tsia
**21.FR-Date de manipulation**<br>*21.EN- Handling date*<br>21.MG- Daty nanaovana azy	**|__|__|/|__|__|/|__|__|__|__| (jj/mm/aaaa)**<br>*|__|__|/|__|__|/|__|__|__|__| (dd/mm/yyyy)*<br>|__|__|/|__|__|/|__|__|__|__| (aa/vv/tttt)
**22.FR-Cartouche Xpert utilisé (Test #1)**<br>*22.EN-* CartridgeXpert used (Test #1)<br>22.MG- ''Cartouche Xpert'' nampiasaina (Test #1)	◯ **MTB/RIF** ◯ **MTB/RIF ULTRA**<br>◯ *MTB/RIF* ◯ *MTB/RIF ULTRA*<br>◯ MTB/RIF ◯ MTB/RIF ULTRA

**33.FR-Résultat semi-quantitatif**<br>*33.EN-semi-quantitative result*<br>33.MG-Vokatra « semi-quantitatif »	◯ **Négatif** ◯ **Trace** ◯ **Très bas** ◯**Bas** ◯ **Moyen** ◯**Elevé** ◯**Invalide** ◯**Erreur** ◯**Pas de résultat**<br>◯ *Negative* ◯ *Trace* ◯ *Very low* ◯*Low* ◯ *Medium* ◯*High* ◯*Invalid* ◯*Error* ◯*No result*<br>◯ Negatifa ◯ Trasy ◯ Tena kely ◯Kely ◯ Antonony ◯Ambony ◯Tsy mety ◯Diso ◯Tsy misy valiny
**FR- Cycle seuil**<br>*EN- Cycle Threshold*<br>MG- Tsingerin'ny ''threshold''	
23.CT-SPC-1	|__|__|.|__|
24.IS 1081-IS 6110-1	|__|__|.|__|
25.CT-RPOB1-1	|__|__|.|__|
26.CT-RPOB2-1	|__|__|.|__|
27.CT-RPOB3-1	|__|__|.|__|
28.CT-RPOB4-1	|__|__|.|__|
**FR- Température maximale de fusion**<br>*EN- Melt peak temperature*<br>MG- Maripana avo indrindra ny fiempoana	
29.Melt-RPOB1-1	|__|__|.|__|
30.Melt -RPOB2-1	|__|__|.|__|
31.Melt -RPOB3-1	|__|__|.|__|
32.Melt -RPOB4-1	|__|__|.|__|
**34.FR- Détection de la résistance à la rifampicine par GeneXpert (Test #1)**<br>*34.EN- Rifampicine resistance detection GeneXpert (Test #1)*<br>34.MG- Fanoherana ny Rifampicine avy amin'ny GeneXpert (Fitiliana #1)	◯ **Non détectée** ◯ **Détectée** ◯ **Indéterminée**<br>◯ *Not detected* ◯ *Detected* ◯ *Undetermined*<br>◯ Tsy nahitana ◯ Nahitana ◯ Tsy voafaritra
**FR - Comme le résultat du 1er test Xpert est '' Invalide, Erreur, Pas de résultat '', le test GeneXpert doit être repris (Test #2)**<br>*EN - Since the 1st Xpert test is "Invalid, Error, No result", the Xpert test needs to be repeated (Test #2)*<br>MG – Raha toa ka «	
**35.FR-Test GeneXpert (Test #2)**<br>*35.EN- GeneXpert test (Test #2)*<br>35.MG- Fitiliana GeneXpert (Test #2)	◯ **Oui** ◯ **Non**<br>◯ Yes ◯ No<br>◯ Eny ◯ Tsia
**36.FR-Date de manipulation**<br>*36.EN- Handling date*<br>36.MG- Daty nanaovana azy	**|__|__|/|__|__|/|__|__|__|__| (jj/mm/aaaa)**<br>*|__|__|/|__|__|/|__|__|__|__| (dd/mm/yyyy)*<br>|__|__|/|__|__|/|__|__|__|__| (aa/vv/tttt
**37.FR-Cartouche Xpert utilisé (Test #2)**<br>*37.EN- CartridgeXpert used (Test #2)*<br>37.MG- ''Cartouche Xpert'' nampiasaina(Test #2)	◯ **MTB/RIF** ◯ **MTB/RIF ULTRA**<br>◯ *MTB/RIF* ◯ *MTB/RIF ULTRA*<br>◯ MTB/RIF ◯ MTB/RIF ULTRA
**48.FR-Résultat semi-quantitatif**<br>*48.EN- Semi-quantitative results*<br>48.MG- Vokatra ''semi-quantitatif''	◯ **Négatif** ◯ **Trace** ◯ **Très bas** ◯ **Bas** ◯ **Moyen** ◯ **Elevé** ◯ **Invalide** ◯ **Erreur** ◯ **Pas de résultat**<br>◯ *Negative* ◯ *Trace* ◯ *Very low* ◯ *Medium* ◯ *High* ◯ *Invalid* ◯ *Error* ◯ *No result*<br>◯ Negatifa ◯ Trasy ◯ Tena kely ◯ Antonony ◯ Ambony ◯ Tsy mety ◯ Diso ◯ Tsisy valiny
**FR- Cycle seuil**<br>*EN-Cycle Threshold*<br>MG- Tsingerin'ny ''threshold''	
38.CT-SPC-1	|__|__|.|__|
39.IS 1081-IS 6110-2	|__|__|.|__|
40.CT-RPOB1-2	|__|__|.|__|
41.CT-RPOB2-2	|__|__|.|__|

42.CT-RPOB3-2	**|__|__|.|__|**
43.CT-RPOB4-2	**|__|__|.|__|**
**FR- Température maximale de fusion**<br>*EN- Melt peak temperature*<br>MG- Maripana avo indrindra ny fiempoana	
44.Melt-RPOB1-2	**|__|__|.|__|**
45.Melt -RPOB2-2	**|__|__|.|__|**
46.Melt -RPOB3-2	**|__|__|.|__|**
47.Melt -RPOB4-2	**|__|__|.|__|**
**49.FR- Détection de la résistance à la rifampicine par GeneXpert (Test #2)**<br>*49.EN- Rifampicine resistance detection GeneXpert (Test #2)*<br>49.MG- Fanoherana ny Rifampicine avy amin'ny GeneXpert (Fitiliana #2)	◯ **Non détectée** ◯ **Détectée** ◯ **Indéterminée**<br>◯ *Not detected* ◯ *Detected* ◯ *Undetermined*<br>◯ Tsy nahitana ◯ Nahitana ◯ Tsy voafaritra
**50.FR-Crachat envoyé à l'IPM ?**<br>*50.EN-Sputum sent to IPM ?*<br>50.MG-Nalefa any amin'ny IPM ve ny santiona-drehoaka ?	◯ **Oui** ◯ **Non**<br>◯ Yes ◯ No<br>◯ Eny ◯ Tsia
**51.FR-Date d'envoi à l'IPM**<br>*51.EN- Date of submission to IPM*<br>51.MG- Daty Nandefasana azy amin'ny IPM	**|__|__|/|__|__|/|__|__|__|__| (jj/mm/aaaa)**<br>*|__|__|/|__|__|/|__|__|__|__| (dd/mm/yyyy)*<br>|__|__|/|__|__|/|__|__|__|__| (aa/vv/tttt)
**52.FR- Numéro de crachat envoyé**<br>*52.EN- Suptum number sent*<br>52.MG- Laharan'ny rehoka nalefa	◯ **1er** ◯ **2ème**<br>◯ *1st* ◯ *2nd*<br>◯ Voalohany ◯ Faharoa
**FR- Le test en microscopie ou le test Xpert est positif. Ceci confirme un diagnsotic de tuberculose.**<br>**→ Aviser le CDT d'appartenance du patient (responsabilité MD coordonateur d'étude)**<br>**→ Acheminer l'échantillon primaire positif à l'IPM**<br>**→ Créer une demande de 3ème échantillon pour envoi direct à l'IPM**<br><br>*EN- The microscopy test or the Xpert test is positive. This confirms a diagnosis of tuberculosis.*<br>*→ Notice the patient's CDT (Study coordinator doctor responsibility)*<br>*→ Forward the positive primary sample to IPM*<br>*→ Create 3rd sample request for direct shipment to IPM*<br><br>MG- Fitiliana microscopie na ny fitiliana Xpert pozitifa. Izany dia manamafy ny fitiliana ny raboka.<br>→ Ampahafantaro ny CDT misy ny marary (mpandrindra ny fianarana MD)<br>→ Alefaso any amin'ny IPM ny santionany voalohany pozitifa<br>→ Mamorona fangatahana santionany faha-3 halefa mivantana any amin'ny IPM	

FR- Résultats de laboratoire
EN- Laboratory results
MG-Vokatry ny labotatoara

**FR- MICROSCOPIE ET CULTURE IPM**<br>*EN- IPM MICROSCOPY AND CULTURE IPM*<br>MG- MIKROSOKOPIA SY FAMBOLENA IMP	
**01.FR-Numéro du crachat**<br>*01.EN-Suptum Number*<br>01.MG- Laharan'ny rehoka	◯ **1er** ◯ **2ème**<br>◯ *1st* ◯ *2nd*<br>◯ Voalohany ◯ Faharoa
**02.FR- Numéro CNRM**<br>*02.EN- CNRM number*<br>02.MG- Nomerao CNRM	**|__|__|__|__|-|__|__|__|__|**<br>*|__|__|__|__|-|__|__|__|__|*<br>|__|__|__|__|-|__|__|__|__|
**03.FR- Date de réception de l'IPM**<br>*03.EN- Date of receipt IPM*<br>03.MG- Daty nandraisan'ny IPM	**|__|__|/|__|__|/|__|__|__|__| (jj/mm/aaaa)**<br>*|__|__|/|__|__|/|__|__|__|__| (dd/mm/yyyy)*<br>|__|__|/|__|__|/|__|__|__|__| (aa/vv/tttt)
**04.FR- Observation qualitatif**<br>*04.EN- Quality observation*<br>04.MG- Fahitana ny kalitao	◯ **Salivaire** ◯ **Purulent** ◯ **Muqueux** ◯ **Mucopurulent** ◯ **Hémoptoique**<br>◯ *Salivary* ◯ *Purulent* ◯ *Mucosal* ◯ *Mucopurulent* ◯ *Hemoptoic*<br>◯ Rora ◯ Nana ◯ Madity ◯ Madity sady misy nana ◯ Misy rà
**05.FR- Volume**<br>*05.EN- Volume*<br>05.MG- Fatra	**|__|__|(mL)**<br>*|__|__| (mL)*<br>|__|__| (mL)
**06.FR-Date de manipulation**<br>*06.EN- Handling date*<br>06.MG- Daty nanaovana azy	**|__|__|/|__|__|/|__|__|__|__| (jj/mm/aaaa)**<br>*|__|__|/|__|__|/|__|__|__|__| (dd/mm/yyyy)*<br>|__|__|/|__|__|/|__|__|__|__| (aa/vv/tttt)
**07.FR- Examen Direct Auramine**<br>*07.EN- Auramin Microscopy*<br>07.MG- Auramine mikroskopia	◯ **Négatif** ◯ **Faible positif** ◯ **1+** ◯ **2+** ◯ **3+** ◯ **Non Fait**<br>**Si " Faible positif ", précisez le nombre de BAAR trouvés |__|__|**<br>◯ *Negative* ◯ *Low positive* ◯ *1+* ◯ *2+* ◯ *3+* ◯ *Not done*<br>*Specify the number of BAAR if ''Low positive'' |__|__|*<br>◯ Tsisy ◯ Misy kely ◯ 1+ ◯ 2+ ◯ 3+ ◯ Tsy natao<br>Raha toa ka ''Misy kely'' dia omeo ny isa hita |__|__|
**FR-Si direct auramine " Faible positif ", précisez le nombre de BAAR trouvés**<br>*EN-If direct auramine 'Low positive', specify the number of BAARs found*<br>MG-Raha toa ka ''Misy kely'' dia omeo ny isa hita	
**08.FR- Date de rendu résultat**<br>*08.EN- Date of results*<br>08.MG- Daty nanolorana ny vokatra	**|__|__|/|__|__|/|__|__|__|__| (jj/mm/aaaa)**<br>*|__|__|/|__|__|/|__|__|__|__| (dd/mm/yyyy)*<br>|__|__|/|__|__|/|__|__|__|__| (aa/vv/tttt)

<table>
<tr><td>09.FR- Culture LJ réalisée
09.EN- Culture LJ done
09.MG- Fambolena LJ natao</td><td>◯ Oui ◯ Non
◯ Yes ◯ No
◯ Eny ◯ Tsia</td></tr>
<tr><td colspan="2">FR- Remplir les champs suivants si la réponse au questionnaire précédent est “”Oui’’
EN-Fill in the following fields if the answer of previous question is “Yes”
MG- Fenoina ireto hefitra manaraka ireto raha toa ka “Eny” no valin’ny fanontaniana eo ambony</td></tr>
<tr><td>10.FR- Date de lancement de la culture LJ
10.EN- Date of culture LJ
10.MG- Daty nanovana ny fambolena tamin’ny LJ</td><td>|__|__|/|__|__|/|__|__|__|__| (jj/mm/aaaa)
|__|__|/|__|__|/|__|__|__|__| (dd/mm/yyyy)
|__|__|/|__|__|/|__|__|__|__| (aa/vv/tttt)</td></tr>
<tr><td>11.FR- Date de résultat LJ
11.EN- Result date LJ
11.MG- Daty ny vokatra</td><td>|__|__|/|__|__|/|__|__|__|__| (jj/mm/aaaa)
|__|__|/|__|__|/|__|__|__|__| (dd/mm/yyyy)
|__|__|/|__|__|/|__|__|__|__| (aa/vv/tttt)</td></tr>
<tr><td>12.FR- Résultat Quantitative
12.EN- Quantitative result
12.MG- Vokatra ‘’Quantitative’’</td><td>◯ 0 ◯1+ ◯ 2+ ◯ 3+ ◯ Positif faible
Si "Positif faible", précisez le nombre de colonies trouvées |__|__|
◯ 0 ◯1+ ◯ 2+ ◯ 3+ ◯ Low Positive
If " Low Positive ", specify the number of colonies found |__|__|
◯ 0 ◯1+ ◯ 2+ ◯ 3+ ◯ Pozitifa ambany
Raha toa ka ‘’positif ambany’, dia farito ny isany |__|__|</td></tr>
<tr><td>FR-Si résultat quantitative "positif faible", précisez le nombre de colonies trouvée
EN-If quantitative "low positive" result, specify the number of colonies found
MG-Raha toa ka ''positif ambany', dia farito ny isany</td><td></td></tr>
<tr><td>13.FR- Culture MGIT réalisée
13.EN- Culture MGIT done
13.MG- Fambolena MGIT natao</td><td>◯ Oui ◯ Non
◯ Yes ◯ No
◯ Eny ◯ Tsia</td></tr>
<tr><td colspan="2">FR- Remplir les champs suivants si la réponse au questionnaire précédent est “”Oui’’
EN-Fill in the following fields if the answer of previous question is “Yes”
MG- Fenoina ireto hefitra manaraka ireto raha toa ka “Eny” no valin’ny fanontaniana eo ambony</td></tr>
<tr><td>14.FR- Date de lancement de la culture MGIT
14.EN- Date of culture MGIT
14.MG- Daty nanovana ny fambolena tamin’ny MGIT</td><td>|__|__|/|__|__|/|__|__|__|__| (jj/mm/aaaa)
|__|__|/|__|__|/|__|__|__|__| (dd/mm/yyyy)
|__|__|/|__|__|/|__|__|__|__| (aa/vv/tttt)</td></tr>
<tr><td>15.FR- Date de résultat MGIT
15.EN- Result date MGIT
15.MG- Daty ny vokatra</td><td>|__|__|/|__|__|/|__|__|__|__| (jj/mm/aaaa)
|__|__|/|__|__|/|__|__|__|__| (dd/mm/yyyy)
|__|__|/|__|__|/|__|__|__|__| (aa/vv/tttt)</td></tr>
<tr><td>16.FR- GU (Unité de croissance)
16.EN- GU (Growth Unit)
16.MG- GU (Taham-pitomboana)</td><td>|________|
|________|
|________|</td></tr>
<tr><td colspan="2" align="center">FR- RETRAITEMENT
EN- REPROCESSING
MG- FAMERENANA NY FAMBOLENA</td></tr>
<tr><td>17.FR- Un retraitement a été réalisé ?
17.EN- Is the reprocessing done?
17.MG- Nanao famerenana ve?</td><td>◯ Oui ◯ Non
◯ Yes ◯ No
◯ Eny ◯ Tsia</td></tr>
<tr><td>18.FR- Date de retraitement
18.EN- Reprocessing date
18.MG- Daty ny Famerenana</td><td>|__|__|/|__|__|/|__|__|__|__| (jj/mm/aaaa)
|__|__|/|__|__|/|__|__|__|__| (dd/mm/yyyy)
|__|__|/|__|__|/|__|__|__|__| (aa/vv/tttt)</td></tr>
<tr><td>19.FR- Date de résultat du retraitement
19.EN- Result date of Reprocessing
19.MG- Daty ny Famerenana</td><td>|__|__|/|__|__|/|__|__|__|__| (jj/mm/aaaa)
|__|__|/|__|__|/|__|__|__|__| (dd/mm/yyyy)
|__|__|/|__|__|/|__|__|__|__| (aa/vv/tttt)</td></tr>
</table>

**20.FR- Résultat du retraitement**<br>*20.EN- Reprocessing result*<br>20.MG- Vokatry ny famerenana	**◯ 0 ◯1+ ◯ 2+ ◯ 3+ ◯ Positif faible**<br>**Si "Positif faible", précisez le nombre de colonies trouvées |__|__|**<br>*◯ 0 ◯1+ ◯ 2+ ◯ 3+ ◯ Low Positive*<br>*If " Low Positive ", specify the number of colonies found |__|__|*<br>◯ 0 ◯1+ ◯ 2+ ◯ 3+ ◯ Pozitifa ambany<br>Rah atoa ka ''positif ambany', dia farito ny isany
**FR-Si résultat quantitative "positif faible", précisez le nombre de colonies trouvée**<br>*EN-If quantitative "low positive" result, specify the number of colonies found*<br>MG-Raha toa ka ''positif ambany', dia farito ny isany	
**21.FR- GU (Unité de croissance)**<br>*21.EN- GU (Growth Unit)*<br>21.MG- GU (Taham-pitomboana)	**|________|**<br>*|________|*<br>|________|
**FR- IDENTIFICATION DE LA SOUCHE**<br>*EN- IDENTIFICATION OF STRAIN*<br>MG- FAMANTARANA NY ''SOUCHE''	
**22.FR- Une identification a été réalisée ?**<br>*22.EN- Is the identification done?*<br>22.MG- Natao ve ny famaritana?	**◯ Oui ◯ Non**<br>*◯ Yes ◯ No*<br>◯ Eny ◯ Tsia
**23.FR- Date de l'identification**<br>*23.EN- Identification date*<br>23.MG- Daty ny famantarana	**|__|__|/|__|__|/|__|__|__|__| (jj/mm/aaaa)**<br>*|__|__|/|__|__|/|__|__|__|__| (dd/mm/yyyy)*<br>|__|__|/|__|__|/|__|__|__|__| (aa/vv/tttt)
**24.FR- Résultat**<br>*24.EN- Result*<br>24.MG- Valiny fitiliana	**◯ CMTB ◯ Atypique ◯ SD_NEG ◯ Non fait**<br>*◯ CMTB ◯ Atypical ◯ SD_NEG ◯ Not done*<br>◯ CMTB ◯ Atypique ◯ SD_NEG ◯ Tsy natao
**25.FR- Si « Atypique », précisez**<br>*25.EN- If "Atypical", specify*<br>25.MG- Raha "Atypical", dia farito	_ _<br>_ _<br>_ _
**FR- BIOBANQUE**<br>*EN- BIOBANK*<br>MG-BIOBANKY	
**26.FR- Est-ce que la souche a été consérvé dans le biobanque ?**<br>*26.EN- Is the biobank done?*<br>26.MG- Natao ve ny biobanky?	**◯ Oui ◯ Non**<br><br>*◯ Yes ◯ No*<br>◯ Eny ◯ Tsia
**27.FR- Date de collection**<br>*27.EN- Collection date*<br>27.MG- Daty nanaovana ny ''collection''	**|__|__|/|__|__|/|__|__|__|__| (jj/mm/aaaa)**<br>*|__|__|/|__|__|/|__|__|__|__| (dd/mm/yyyy)*<br>|__|__|/|__|__|/|__|__|__|__| (aa/vv/tttt)
**28.FR- OPTIM du congélateur**<br>*28.EN- Freezer OPTIM*<br>28.MG- OPTIM ny vata fampangatsiahana	
**29.FR- Numéro de la boîte**<br>*29.EN- Box number*<br>29.MG- Nomerao ny boaty	

<table>
<tr><td>30.FR- 3ème crachat
30.EN- 3rd sputum
30.MG- Rehoka fahatelo</td><td>◯ Oui ◯ Non
◯ Yes ◯ No
◯ Eny ◯ Tsia</td></tr>
<tr><td colspan="2">FR- Remplir les champs suivants si la réponse au questionnaire précédent est “”Oui’’
EN-Fill in the following fields if the answer of previous question is “Yes”
MG- Fenoina ireto hefitra manaraka ireto raha toa ka “Eny” no valin’ny fanontaniana eo ambony</td></tr>
<tr><td>31.FR- Numéro CNRM
31.EN-CNRM Number
31.MG- Numerao CNRM</td><td>|__|__|__|__|-|__|__|__|__|
|__|__|__|__|-|__|__|__|__|
|__|__|__|__|-|__|__|__|__|</td></tr>
<tr><td>32.FR- Date de réception de l’IPM
32.EN- Date of receipt IPM
32.MG- Daty nandraisan’ny IPM</td><td>|__|__|/|__|__|/|__|__|__|__| (jj/mm/aaaa)
|__|__|/|__|__|/|__|__|__|__| (dd/mm/yyyy)
|__|__|/|__|__|/|__|__|__|__| (aa/vv/tttt)</td></tr>
<tr><td>33.FR- Observation qualitatif
33.EN- Quality observation
33.MG- Fahitana ny kalitao</td><td>◯ Salivaire ◯ Purulent ◯ Muqueux ◯ Mucopurulent ◯ Hémoptoique
◯ Salivary ◯ Purulent ◯ Mucosal ◯ Mucopurulent ◯ Hemoptoic
◯ Rora ◯ Nana ◯ Madity ◯ Madity sady misy nana ◯ Misy rà</td></tr>
<tr><td>34.FR- Volume
34.EN- Volume
34.MG- Fatra</td><td>|__|__|(mL)
|__|__| (mL)
|__|__| (mL)</td></tr>
<tr><td>35.FR-Date de manipulation
35.EN- Handling date
35.MG- Daty nanaovana azy</td><td>|__|__|/|__|__|/|__|__|__|__| (jj/mm/aaaa)
|__|__|/|__|__|/|__|__|__|__| (dd/mm/yyyy)
|__|__|/|__|__|/|__|__|__|__| (aa/vv/tttt)</td></tr>
<tr><td>36.FR- Examen Direct Auramine
36.EN- Auramin Microscopy
36.MG- Auramine mikroskopia</td><td>◯ Négatif ◯ Faible positif ◯ 1+ ◯ 2+ ◯ 3+ ◯ Non Fait
Si " Faible positif ", précisez le nombre de BAAR trouvés |__|__|
◯ Negative ◯ Low positive ◯ 1+ ◯ 2+ ◯ 3+ ◯ Not done
Specify the number of BAAR if ‘’Low positive’’ |__|__|
◯ Tsisy ◯ Misy kely ◯ 1+ ◯ 2+ ◯ 3+ ◯ Tsy natao
Raha toa ka ‘’Misy kely’’ dia omeo ny isa hita |__|__|</td></tr>
<tr><td>FR-Si résultat "positif faible", précisez le nombre de colonies trouvée
EN-If result "low positive" result, specify the number of colonies found
MG-Raha toa ka ''positif ambany', dia farito ny isany</td><td></td></tr>
<tr><td>37.FR- Date de rendu résultat
37.EN- Date of results
37.MG- Daty nanolorana ny vokatra</td><td>|__|__|/|__|__|/|__|__|__|__| (jj/mm/aaaa)
|__|__|/|__|__|/|__|__|__|__| (dd/mm/yyyy)
|__|__|/|__|__|/|__|__|__|__| (aa/vv/tttt)</td></tr>
<tr><td>38.FR- Culture LJ réalisée
38.EN- Culture LJ done
38.MG- Fambolena LJ natao</td><td>◯ Oui ◯ Non
◯ Yes ◯ No
◯ Eny ◯ Tsia</td></tr>
<tr><td colspan="2">FR- Remplir les champs suivants si la réponse au questionnaire précédent est “”Oui’’
EN-Fill in the following fields if the answer of previous question is “Yes”
MG- Fenoina ireto hefitra manaraka ireto raha toa ka “Eny” no valin’ny fanontaniana eo ambony</td></tr>
<tr><td>39.FR- Date de lancement de la culture LJ
39.EN- Date of culture LJ
39.MG- Daty nanovana ny fambolena tamin’ny LJ</td><td>|__|__|/|__|__|/|__|__|__|__| (jj/mm/aaaa)
|__|__|/|__|__|/|__|__|__|__| (dd/mm/yyyy)
|__|__|/|__|__|/|__|__|__|__| (aa/vv/tttt)</td></tr>
<tr><td>40.FR- Date de résultat LJ
40.EN- Result date LJ
40.MG- Daty ny vokatra</td><td>|__|__|/|__|__|/|__|__|__|__| (jj/mm/aaaa)
|__|__|/|__|__|/|__|__|__|__| (dd/mm/yyyy)
|__|__|/|__|__|/|__|__|__|__| (aa/vv/tttt)</td></tr>
</table>

<table>
<tr><td>41.FR- Résultat Quantitative
41.EN- Quantitative result
41.MG- Vokatra ‘’Quantitative’’</td><td>◯ 0 ◯1+ ◯ 2+ ◯ 3+ ◯ Positif faible
Si "Positif faible", précisez le nombre de colonies trouvées |__|__|
◯ 0 ◯1+ ◯ 2+ ◯ 3+ ◯ Low Positive
If " Low Positive ", specify the number of colonies found |__|__|
◯ 0 ◯1+ ◯ 2+ ◯ 3+ ◯ Pozitifa ambany
Raha toa ka ‘’positif ambany’, dia farito ny isany |__|__|</td></tr>
<tr><td>FR-Si résultat quantitative "positif faible", précisez le nombre de colonies trouvée
EN-If quantitative "low positive" result, specify the number of colonies found
MG-Raha toa ka ''positif ambany', dia farito ny isany</td><td></td></tr>
<tr><td>42.FR- Culture MGIT réalisée
42.EN- Culture MGIT done
42.MG- Fambolena MGIT natao</td><td>◯ Oui ◯ Non
◯ Yes ◯ No
◯ Eny ◯ Tsia</td></tr>
<tr><td colspan="2">FR- Remplir les champs suivants si la réponse au questionnaire précédent est “”Oui’’
EN-Fill in the following fields if the answer of previous question is “Yes”
MG- Fenoina ireto hefitra manaraka ireto raha toa ka “Eny” no valin’ny fanontaniana eo ambony</td></tr>
<tr><td>43.FR- Date de lancement de la culture MGIT
43.EN- Date of culture MGIT
43.MG- Daty nanovana ny fambolena tamin’ny MGIT</td><td>|__|__|/|__|__|/|__|__|__|__| (jj/mm/aaaa)
|__|__|/|__|__|/|__|__|__|__| (dd/mm/yyyy)
|__|__|/|__|__|/|__|__|__|__| (aa/vv/tttt)</td></tr>
<tr><td>44.FR- Date de résultat MGIT
44.EN- Result date MGIT
44.MG- Daty ny vokatra</td><td>|__|__|/|__|__|/|__|__|__|__| (jj/mm/aaaa)
|__|__|/|__|__|/|__|__|__|__| (dd/mm/yyyy)
|__|__|/|__|__|/|__|__|__|__| (aa/vv/tttt)</td></tr>
<tr><td>45.FR- GU (Unité de croissance)
45.EN- GU (Growth Unit)
45.MG- GU (Taham-pitomboana)</td><td>|________|
|________|
|________|</td></tr>
<tr><td colspan="2" align="center">FR- RETRAITEMENT
EN- REPROCESSING
MG- FAMERENANA NY FAMBOLENA</td></tr>
<tr><td>46.FR- Un retraitement a été réalisé ?
46.EN- Is the reprocessing done?
46.MG- Nanao famerenana ve?</td><td>◯ Oui ◯ Non
◯ Yes ◯ No
◯ Eny ◯ Tsia</td></tr>
<tr><td>47.FR- Date de retraitement
47.EN- Reprocessing date
47.MG- Daty ny Famerenana</td><td>|__|__|/|__|__|/|__|__|__|__| (jj/mm/aaaa)
|__|__|/|__|__|/|__|__|__|__| (dd/mm/yyyy)
|__|__|/|__|__|/|__|__|__|__| (aa/vv/tttt)</td></tr>
<tr><td>48.FR- Date de résultat du retraitement
48.EN- Result date of Reprocessing
48.MG- Daty ny Famerenana</td><td>|__|__|/|__|__|/|__|__|__|__| (jj/mm/aaaa)
|__|__|/|__|__|/|__|__|__|__| (dd/mm/yyyy)
|__|__|/|__|__|/|__|__|__|__| (aa/vv/tttt)</td></tr>
<tr><td>49.FR- Résultat du retraitement
49.EN- Reprocessing result
49.MG- Vokatry ny famerenana</td><td>◯ 0 ◯1+ ◯ 2+ ◯ 3+ ◯ Positif faible
Si "Positif faible", précisez le nombre de colonies trouvées |__|__|
◯ 0 ◯1+ ◯ 2+ ◯ 3+ ◯ Low Positive
If " Low Positive ", specify the number of colonies found |__|__|
◯ 0 ◯1+ ◯ 2+ ◯ 3+ ◯ Pozitifa ambany
Rah atoa ka ‘’positif ambany’, dia farito ny isany |__|__|</td></tr>
</table>

**FR-Si résultat quantitative "positif faible", précisez le nombre de colonies trouvée**<br>*EN-If quantitative "low positive" result, specify the number of colonies found*<br>MG-Raha toa ka ''positif ambany', dia farito ny isany	
**50.FR- GU (Unité de croissance)**<br>*50.EN- GU (Growth Unit)*<br>**50.MG- GU (Taham-pitomboana)**	**|________|**<br>*|________|*<br>|________|
**FR- IDENTIFICATION DE LA SOUCHE**<br>*EN- IDENTIFICATION OF STRAIN*<br>MG- FAMANTARANA NY ''SOUCHE''	
**51.FR- Une identification a été réalisée ?**<br>*51.EN- Is the identification done?*<br>51.MG- Natao ve ny famaritana?	◯ **Oui** ◯ **Non**<br>◯ *Yes* ◯ *No*<br>◯ Eny ◯ Tsia
**52.FR- Date de l'identification**<br>*52.EN- Identification date*<br>52.MG- Daty ny famantarana	**|__|__|/|__|__|/|__|__|__|__| (jj/mm/aaaa)**<br>*|__|__|/|__|__|/|__|__|__|__| (dd/mm/yyyy)*<br>|__|__|/|__|__|/|__|__|__|__| (aa/vv/tttt)
**53.FR- Résultat**<br>*53.EN- Result*<br>53.MG- Valin'ny fitiliana	◯ **CMTB** ◯ **Atypique** ◯ **SD_NEG** ◯ **Non fait**<br>◯ *CMTB* ◯ *Atypical* ◯ *SD_NEG* ◯ *Not done*<br>◯ CMTB ◯ Atypique ◯ SD_NEG ◯ Tsy natao
**54.FR- Si « Atypique », précisez**<br>*54.EN- If "Atypical", specify*<br>54.MG- Raha "Atypical", dia farito	_ _<br>_ _<br>_ _
**FR- BIOBANQUE**<br>*EN- BIOBANK*<br>MG-BIOBANKY	
**55.FR- Est-ce que la souche a été consérvé dans le biobanque ?**<br>*55.EN- Is the biobank done?*<br>55.MG- Natao ve ny biobanky?	◯ **Oui** ◯ **Non**<br>◯ *Yes* ◯ *No*<br>◯ Eny ◯ Tsia
**56.FR- Date de collection**<br>*56.EN- Collection date*<br>56.MG- Daty nanaovana ny ''collection''	**|__|__|/|__|__|/|__|__|__|__| (jj/mm/aaaa)**<br>*|__|__|/|__|__|/|__|__|__|__| (dd/mm/yyyy)*<br>|__|__|/|__|__|/|__|__|__|__| (aa/vv/tttt)
**57.FR- OPTIM du congélateur**<br>*57.EN- Freezer OPTIM*<br>57.MG- OPTIM ny vata fampangatsiahana	
**58.FR- Numéro de la boîte**<br>*58.EN- Box number*<br>58.MG- Nomerao ny boaty	

FR – Consentement libre et éclairé
EN – Informed consent
MG- *Fanekena an-tsitrapo*

**FR - CONSENTEMENT**<br>*EN - CONSENT*<br>MG - FANEKENA	
**01.FR – Le patient consent à participer à l'étude**<br>*01.EN – The patient consents to participating in the study*<br>01.MG -Manaiky ny andray anjara amin'ny fikarohana ve ?	◯ **Oui** ◯ **Non**<br>◯ *Yes* ◯ *No*<br>◯ Eny ◯ Tsia
**02.FR – Le formulaire de consentement est signé**<br>*02.EN – The informed consent for mis signed*<br>02.MG – Voasonia ny taratasy fanekena an-tsitrapo	◯ **Oui** ◯ **Non**<br>◯ *Yes* ◯ *No*<br>◯ Eny ◯ Tsia
**03.FR - Date de signature du consentement**<br>*03.EN - Date* of signature of consent<br>03.MG – Daty nanaovana ny sonia ny taratasy fanekena	**|__|__| / |__|__| / |__|__|__|__| (jj/mm/aaaa)**<br>*|__|__| / |__|__| / |__|__|__|__| (dd/mm/yyyy)*<br>|__|__| / |__|__| / |__|__|__|__| *(aa/vv/tttt)*

FR-Mesures opérationelle-Séquençage d'ADN et résultats

EN- gDNA sequencing operational metrics and results

MG- Asa natao sy vokatry ny ''Séquençage d'ADN''

<table>
<tr><th colspan="2">IDENTIFICATION</th></tr>
<tr><td>01.FR-Numéro d'identification unique de l'étude
01.EN-ID number of partcipant
01.MG-Laharam'pamantarana ny mpandray anjara</td><td></td></tr>
<tr><td>02.FR – Date de naissance
02.EN – Birthdate
02.MG – Daty nahaterahana</td><td>|__|__| / |__|__| / |__|__|__|__| (jj/mm/aaaa)
|__|__| / |__|__| / |__|__|__|__| (dd/mm/yyyy)
|__|__| / |__|__| / |__|__|__|__| (aa/vv/tt)</td></tr>
<tr><td>03.FR-Genre
03.EN-Gender
03.MG-Fananahana</td><td>◯masculin ◯féminin
◯Male ◯Female
◯lahy ◯vavy</td></tr>
<tr><td>04.FR-Nom du Fokontany
04.EN-Name of Fokontany
04.MG-Anaran'ny Fokontany</td><td></td></tr>
<tr><th colspan="2">EXTRACTION ET RESULTATS DU WGS</th></tr>
<tr><td>05.FR- Numéro Crachat
05.EN- Suptum Number
05.MG- Rehoka</td><td>◯ 1er ◯ 2ème ◯ 3ème
◯ 1st ◯ 2nd ◯ 3rd
◯ Voalohany ◯ Faharoa ◯ Fahatelo</td></tr>
<tr><td>06.FR- Date extraction
06.EN - Date of extraction
06.MG - Daty ny fitrandrahana</td><td>|__|__|/|__|__|/|__|__|__|__| (jj/mm/aaaa)
|__|__|/|__|__|/|__|__|__|__| (dd/mm/yyyy)
|__|__|/|__|__|/|__|__|__|__| (aa/vv/tttt)</td></tr>
<tr><td>07.FR- Milieu de culture
07.EN-Culture
07.MG- « Culture » ampesaina</td><td>◯ MGIT ◯ LJ
◯ MGIT ◯ LJ
◯ MGIT ◯ LJ</td></tr>
<tr><td>08.FR- Méthode d'extraction
08.EN- Extraction method
08.MG- Fomba fitrandrahana</td><td>◯Ethanol_precipitation/ ◯Bille_ampure/ ◯Van_Embden / ◯UCP_kit
◯Ethanol_precipitation/ ◯Bille_ampure/ ◯Van_Embden / ◯UCP_kit</td></tr>
</table>

<table>
<tr><td></td><td>◯Ethanol_precipitation/ ◯Bille_ampure/ ◯Van_Embden / ◯UCP_kit</td></tr>
<tr><td>09.FR- Qubit
09.EN- Qubit
09.MG- Qubit</td><td>|________| (ng/µL)
|________| (ng/µL)
|________| (ng/µL)</td></tr>
<tr><td>10.FR- Nanodrop
10.EN-Nanodrop
10.MG-Nanodrop</td><td>|________| (ng/µL)
|________| (ng/µL)
|________| (ng/µL)</td></tr>
<tr><td>11.FR- Rapport 260/280
11.EN- Ratio 260/280
11.MG- Tatitra 260/280</td><td>|__|__|.|__|__|
|__|__|.|__|__|
|__|__|.|__|__|</td></tr>
<tr><td>12.FR- Rapport 260/230
12.EN- Ratio 260/230
12.MG- Tatitra 260/230</td><td>|__|__|.|__|__|
|__|__|.|__|__|
|__|__|.|__|__|</td></tr>
<tr><td>13.FR- Date de préparation librairie
13.EN- Library preparartion date
13.MG- Daty nanaovana ny ‘’librairie ‘’</td><td>|__|__|/|__|__|/|__|__|__|__| (jj/mm/aaaa)
|__|__|/|__|__|/|__|__|__|__| (dd/mm/yyyy)
|__|__|/|__|__|/|__|__|__|__| (aa/vv/tttt)</td></tr>
<tr><td>14.FR- Date de séquençage
14.EN- Sequencing date
14.MG- Daty nanaovana ny ‘’séquençage’’</td><td>|__|__|/|__|__|/|__|__|__|__| (jj/mm/aaaa)
|__|__|/|__|__|/|__|__|__|__| (dd/mm/yyyy)
|__|__|/|__|__|/|__|__|__|__| (aa/vv/tttt)</td></tr>
<tr><td>15.FR-Numéro de barcode
15.EN-Barcode number
15.MG-Laharan’ny barcode</td><td></td></tr>
<tr><td>FR-IPELINE MYKROBE/TBPORE
EN-IPELINE MYKROBE/TBPORE
MG-IPELINE MYKROBE/TBPORE</td><td></td></tr>
<tr><td>16.FR-Groupe Phylogénétique
16.EN-Phylogenetic group
16.MG-Vondrona filojenika</td><td></td></tr>
<tr><td>17.FR-Phylo couverture %
17.EN-Phylo cover %
17.MG-Phylo couverture %</td><td></td></tr>
<tr><td>18.FR-Phylo profondeur médiane
18.EN-Phylo median depth
18.MG- Phylo profondeur médiane</td><td></td></tr>
<tr><td>19.FR—Espèce
19.EN-Specie
19.MG-Karazana</td><td></td></tr>
<tr><td>20.FR-Espèce couverture %
20.EN-Specie cover %
20.MG-Espèce couverture %</td><td></td></tr>
<tr><td>21.FR-Espèce profondeur médiane
21.EN-Specie median depth
21.MG-Espèce profondeur médiane</td><td></td></tr>
<tr><td>22.FR-Lignée
22.EN-Lineage
22.MG-Lignée</td><td></td></tr>
</table>

**23.FR-Résistance aux Antibiotiques**<br>*23.EN-Antibiotic resistance*<br>23.MG-	
**24-Nombre reads**<br>*24-Number of reads*<br>24.MG-	
**25.FR-Taille/reads moyenne**<br>*25.EN-Medium size/reads*<br>25.MG-	
**26.FR-Bases totales**<br>*26.EN-Total basis*<br>26.MG-	
**27.FR-Qualité moyenne des reads**<br>*27.EN-Average quality of reads*<br>27.MG-	
**28.FR-Date de lancement pipeline**<br>*28.EN-Date of pipeline launch*<br>28.MG- Daty nanombohan'ny Pipeline	
**29.FR-SNPs par rapport au référence**<br>*29.EN-SNPs compared to the reference*<br>29.MG-SNPs ampitahana amin'ny reference	
**30.FR- Présence de cluster**<br>*30.EN- Cluster presence*<br>30.MG- Fisian'ny ''Cluster''	◯ **Oui** ◯ **Non**<br>◯ *Yes* ◯ *No*<br>◯ Eny ◯ Tsia
**31.FR- Identifiants des patients présentant une souche apparentée**<br>*31.EN- Patient identified with a related strain*<br>31.MG- Famantarana manokana ireo marary mitovy fotony	
**FR-Nombre de SNPs de différence**<br>*EN-Number of SNPs of difference*<br>MG-Isan'ny SNPs	
**32.FR-Numero du cluster dans l'étude**<br>*32.EN-Cluster number in the study*<br>32.MG-Laharan'ny cluster	

**33.FR- Date rendu des résultat WGS**<br>*33.EN- Date of WGS results*<br>33.MG- Daty nanolorana ny vokatry ny WGS	**|__|__|/|__|__|/|__|__|__|__| (jj/mm/aaaa)**<br>*|__|__|/|__|__|/|__|__|__|__| (dd/mm/yyyy)*<br>|__|__|/|__|__|/|__|__|__|__| (aa/vv/tttt)
**34.FR- Nom de la personne qui a reçu les résultats**<br>*34.EN- Name of the recipient of the results.*<br>34.MG- Anaran'ny olona nandray ireo vokatra	

FR – Investigation domiciliaire

EN – Domiciliary investigation

MG-fitadiavana ny tranga ao an-tokatrano

**FR – RECHERCHE DE CAS DOMICILIAIRES**<br>*EN – DOMICILIARY CASDES FINDING*<br>MG -Fitadiavana ny tranga ao an-tokantrano	
**01.FR - Indépendamment de votre adresse, y a-t-il quelqu'un qui vit sous le même toit que vous ou qui a vécu avec vous de façon prolongé durant les dernier 3 mois?**<br>*01.EN - Regardless of your address, is there anyone who lives in the same household as you or who has lived with you for a prolonged period of time during the past 3 months?*<br>01.MG - Nisy olona ve miara-mipetraka aminao na niara-nipetraka maharitra taminao tao antin'izay 3 volana izay ? (tsy miankina amin'ny toerana ipetrahana)	◯**Oui** ◯**Non**<br>◯*Yes* ◯*No*<br>◯Eny ◯Tsia
**02.FR – Nombre de ces personnes**<br>*02.EN – Number of those persons*<br>02.MG – Firy ny isan'ireo olona ireo	**|__|__|**<br>*|__|__|*<br>|__|__|

Institut Pasteur de Madagascar / CRCHUM Centre de recherche, Centre hospitalier de l'Université de Montréal	**TB WGS cRCT Haute Matsiatra**<br>*« Séquençage du Génome Complet de la Tuberculose pour le Contrôle de la Maladie à Madagascar - Un Essai Contrôlé Randomisé en Grappe pour Évaluer Différentes Stratégies d'Intervention à l'Échelle Communautaire »*<br><br>**CAHIER D'OBSERVATION**<br>**CRF6.1_WGScRCT_V2_2022.04.25**

FR-LISTE INDIVIDUS CID

EN-LIST OF INDIVIDUALS CID

MG-LISITRY IREO OLONA NIFANDRAISANA

**FR –RECHERCHE DE CAS DOMICILIARES**<br>*EN –DOMICILIARY CASE FINDING*<br>MG –FITADIAVANA NY TRANGA AO AN-TOKATRANO	
**01.FR - Nom**<br>*01.EN - Last name*<br>01.MG - Anarana	
**02.FR - Prénoms**<br>*02.EN - First name*<br>02.MG - Fanampin'anarana	
**03.FR - Age**<br>03.EN - Age<br>*03.MG - taona*	**|__|__|**<br>*|__|__|*<br>|__|__|
**04.FR - Genre**<br>*04.EN - Gender*<br>04.MG - Fananahana	◯**Masculin** ◯**Féminin**<br>◯*Male* ◯*Female*<br>◯lahy ◯Vavy
**05.FR - Type de contact**<br>*05.EN - Type of contact*<br>*05.MG – Karazana fifandraisana*<br><br>**Étroit= contact permanent**<br>**Régulier= contact de plus de 50% des nuités**<br>**Occasionnel=contact moins de 50% des nuités**<br>*Close= permanent contact with the index case*<br>*Regular= contact more than 50% of nights*<br>*Occasional= contact less than 50% of nights*<br>Akaiky= olona mifandray maharitra<br>Matetika= olona mifandray mihoatra ny 50% amin'ny alina<br>Indraindray= olona mifandray latsaky ny 50% amin'ny alina	◯**Étroit** ◯**Régulier** ◯**Occasionnel**<br>◯*Closed* ◯*Regular* ◯*Occasional*<br>◯Akaiky ◯Matetika ◯Indraindray

	**TB WGS cRCT Haute Matsiatra**<br>*« Séquençage du Génome Complet de la Tuberculose pour le Contrôle de la Maladie à Madagascar - Un Essai Contrôlé Randomisé en Grappe pour Évaluer Différentes Stratégies d'Intervention à l'Échelle Communautaire »*<br><br>**CAHIER D'OBSERVATION**<br>**CRF7_WGScRCT_V3_2022.12.01**

FR – Investigation épidémiologique d'agrégats

EN – Cluster epidemiological investigation

MG- Fanadihadiana epidemiolojika momba ny vondrona

**CRF 7.0**	**FR - IDENTIFICATION**<br>*EN - IDENTIFICATION*<br>MG - FAMANTARANA
**01.FR-Numéro de dossier du participant**<br><br>*01.EN-Participant file number*<br><br>01.MG-Laharan'ny dosie ny mpandray anjara	**DE/ID/EP|__|__|__|__|__|**<br><br>*DE/ID/EP |__|__|__|__|__|*<br><br>DE/ID/EP |__|__|__|__|__|
**02.FR - Identité de la personne qui remplit le questionnaire**<br>*02.EN- Identity of the research personnel filling the questionnaire*<br>02.MG- Famantarana ny olona mameno ny andiam-panontaniana	
**FR – INFORMATIONS DE BASE SUR L'AGRÉGAT**<br>*EN – CLUSTER BASE INFORMATION*<br>MG – MOMBAMOMBA NY VONDRONA	
**03.FR – S'agit-il d'un nouvel agrégat ?**<br>*03.EN- Is this a new cluster ?*<br>03.MG-Vondrona vaovao ve ?	◯ **Oui nouvel agrégat** ◯ **Non agrégat connu**<br>◯ *Yes new cluster* ◯ *No known cluster*<br>◯ Eny vondrona vaovao ◯*Tsia vondrona efa misy*
**04.FR - Numéro de l'agrégat en investigation**<br>*04.EN – Investigated cluster number*<br>04.MG – Laharanan'ny vondrona ao anatin'ny fanadihadiana	**C |__|__|__|__|**<br>*C |__|__|__|__|*<br>C |__|__|__|__|
**05.FR - Date du début de l'investigation de cet agrégat**<br>*05.EN – Initial start date of this cluster investigation*<br>05.MG -Daty nanombohan'ny fanadihadina ao anatin'ny vondrona	**|__|__| / |__|__| / |__|__|__|__| (jj/mm/aaaa)**<br>*|__|__| / |__|__| / |__|__|__|__| (dd/mm/yyyy)*<br>|__|__| / |__|__| / |__|__|__|__| (aa/vv/tttt)
**06.FR - Date de la présente investigation**<br>*06.EN – Current investigation date*<br>06.MG – Daty ny fanadihadiana ny vondrona izao	**|__|__| / |__|__| / |__|__|__|__| (jj/mm/aaaa)**<br>*|__|__| / |__|__| / |__|__|__|__| (dd/mm/yyyy)*<br>|__|__| / |__|__| / |__|__|__|__| (aa/vv/tttt)
**07. Différence de SNPs intra-cluster**<br>*07.EN-SNPs difference in the cluster*<br>07.MG-Fahasamihafan'ny SNP ao anaty vondrona	

FR-Pour chaque participant inclus dans l'agrégat
EN-For each participant involved in the cluster
MG-Ho an'ny mpandray anjara tafiditra ao anaty vondrona

**CRF 7.1** **FR-LISTE DES PARTICIPANTS INCLUS DANS L'AGREGAT**<br>*EN- LIST OF PARTICIPANTS INVOLVED IN THE CLUSTER*<br>MG- LISITR'IREO MPANDRAY ANJARA TAFIDITRA AO ANATY VONDRONA	
**01.FR-Identifiant unique du patient inclus dans cet agrégat**<br>*01.EN-ID number of the patient involved in the cluster*<br>01.MG-Lahara-pamantarana ny mpandray anjara tafiditra ao anaty vondrona	**PERS |__|__|__|__|__|**<br>*PERS |__|__|__|__|__|*<br>PERS |__|__|__|__|__|
**02.FR – Fokontany du patient impliqué dans cet agrégat**<br>*02.EN – First name of patient involved in this cluster*<br>02.MG – Fokontany misy ny mpandray anjara tafiditra ao anatin'ity vondrona ity	
**03.FR – Village du patient impliqué dans cet agrégat**<br>*03.EN – Village of patient involved in this outbreak*<br>03.MG – Vondrotrano misy ny mpandray anjara tafiditra ao anatin'ity vondrona ity	
**04.FR – Présence de lien domiciliaire potentiels déjà connus**<br>*04.EN – Previsouly known potential household links*<br>04.MG -Mety misy fifandraisana ao an-tokatrano ve ?<br><br>**FR-Si la réponse est « non », renvoi à la question n°7**<br>*EN- If the answer is « No », refer to the question n°7*<br>MG-Raha toa ka « tsia » ny valiny, tohizana avy hatrany amin'ny fanontaniana n°7	◯**Oui** ◯**Non**<br>◯*Yes* ◯*No*<br>◯Eny ◯Tsia
**FR - ENQUÊTE TERRAIN**<br>*EN - FIELD INVESTIGATION*<br>MG – FANADIHADIANA ENY IFOTONY	
**05.FR – Est-ce que les liens domiciliaires potentiels sont confirmés**<br>*05.EN – Are potential household links confirmed*<br>05.MG – Voamarina ve ny fifandraisana ao an-tokatrano ?<br><br>**FR-Si la réponse est « non », renvoi à la question n°7**<br>*EN- If the answer is « No », refer to the question n°7*<br>MG-Raha toa ka « tsia » ny valiny, tohizana avy hatrany amin'ny fanontaniana n°7	◯**Oui** ◯**Non**<br>◯*Yes* ◯*No*<br>◯Eny ◯Tsia
**06.FR – Décrivez les liens domiciliaires confirmés**<br>*06.EN – Describe confirmed household links*<br>06.MG – Farito ny fifandraisana ao antokatrano	
**07.FR – Est-ce que les patients impliqués dans l'agrégat se connaissent**<br>*07.EN – Do patients involved in the cluster know each other*<br>07.MG -Mifankafantatra ve ny olona ao anatin'ny vondrona ?	◯**Oui** ◯**Non**<br>◯*Yes* ◯*No*<br>◯Eny ◯Tsia

**08.FR – Décrivez les liens identifiés**<br>*08.EN – Describe identified links*<br>08.MG -Farito ny fifandraisana misy amin'izy ireo	
**09.FR –Quels sont les lieux visités sur base quotidienne ? (ex. maison, champ, école…)**<br>*09.EN – Which places are visited on a daily basis? (ex. house, field, school…)*<br>09.MG -Farito ny toerana falehany isan'andro (trano, eny an-tsaha, toeram-pianarana…)	
**10.FR – Lequels sont les lieux visités sur base occasionnelle ?**<br>**(ex. église, marché, école…)**<br>*10.EN – Which places are visited on occasion? (ex. church, market, school…)*<br>10.MG -Farito ny toerana falehany indraindray<br>(oh : fiangonana, tsena, toeram-pianarana…)	
**11.FR –Quand sont ces lieux visités ?**<br>*11.EN –When are those physical location visited?*<br>11.MG -Ovina no nandeha tamin'ireo toerana ireo ?	

**CRF 7.2**	**FR-INVESTIGATION EPIDEMIOLOGIQUE DE L'AGREGAT**<br>*EN- EPIDEMIOLOGICAL INVESTIGATION OF THE CLUSTER*<br>MG-FANADIHADIANA ARA EPIDEMIOLOJIKA NY VONDRONA
**01.FR – Est-ce qu'un lien spatiotemporel entre les patients de l'agrégat peut être établi ?**<br>*01.EN – Can a spatiotemporal link between cluster patients be established?*<br>01.MG -Mety misy fifandraisana ara-potoana sy toerana ve ny mpandray anjara ao anatin'ny vondrona ?<br>**FR-Si la réponse est « non », fin de questionnaires.**<br>*EN- If the answer is « no », end of questionnaires*.<br>MG- Raha toa ka « tsia » ny valiny, mifarana ny fanontaniana.	◯**Oui** ◯**Non**<br>◯*Yes* ◯*No*<br>◯Eny ◯Tsia
**02.FR – Décrivez le lien spatiotemporel entre les patients de l'agrégat ?**<br>*02.EN – Describe the spatiotemporal link between patients in the cluster*<br>02.MG -Farito ny fifandraisana ara-potoana sy toerana, misy amin'ireo mpandray anjara ao anatin'ny vondrona	
**03.FR – Quel stratégie de dépistage complémentaire peut être établie pour diagnostiquer des cas additionnels dans cet agrégat?**<br>*03.EN – Which screening strategy can be implemented to diagnose additional cases within this cluster?*<br>03.MG - Inona no paikadin'ny fitiliana fanampiny azo apetraka hamantarana ny tranga fanampiny amin'ity vondrona ity	

<table>
<tr><td>Institut Pasteur de Madagascar
CRCHUM
CENTRE DE RECHERCHE
Centre hospitalier de l'Université de Montréal</td><td>UNITE D'EPIDEMIOLOGIE ET RECHERCHE CLINIQUE

« Séquençage du Génome Complet de la Tuberculose pour le Contrôle de la Maladie à Madagascar - Un Essai Contrôlé Randomisé en Grappe pour Évaluer Différentes Stratégies d'Intervention à l'Échelle Communautaire »

TB WGS cRCT
CRF8.0_WGScRCT_V2_2023.05.11</td></tr>
</table>

<table>
<tr><td colspan="2">FR-IDENTIFICATION
EN-IDENTIFICATION
MG-FAMANTARANA</td></tr>
<tr><td>FR-Numéro de dossier du participant
EN-Participant file number
MG-Laharan'ny dosie ny mpandray anjara</td><td>(DE ou ID ou EP)|__|__|__|__|__|
(DE or ID or EP)|__|__|__|__|__|
(DE na ID na EP)|__|__|__|__|__|</td></tr>
<tr><td>01.FR-Date de remplissage
01.EN-Date of filling
01.MG-Daty amenoana ny adiam-panotaniana</td><td>|__|__| / |__|__| / |__|__|__|__| (jj/mm/aaaa)
|__|__| / |__|__| / |__|__|__|__| (dd/mm/yyyy)
|__|__| / |__|__| / |__|__|__|__| (aa/vv/tttt)</td></tr>
<tr><td>02.FR-IRC responsable
02.EN-IRC responsible
02.MG-IRC tompon'andraikitra</td><td></td></tr>
<tr><td>09.FR-Est-ce un tuberculeux diagnostiqué lors de la V1 ?
09.EN- tuberculosis patient diagnosed in V1 ?
09.MG- Mpandray anjara voatily mitondra raboka tao amin'ny V1?

FR- Si la réponse est « non », renvoi à la Q°11
EN- If the answer is "no", refer to Q°11
MG-Raha « Tsia » ny valiny, tohizana amin'ny fanontaniana n°11</td><td>◯Oui ◯Non</td></tr>
<tr><td>10.FR-Si oui, est ce que le participant a été revu lors de la V2 ?
10.EN- If so, was the participant reviewed during V2?
10.MG-Raha eny, efa noverenana nohadihadiana ve ny mpandray anjara nandritra ny V2 ?

FR-Si la réponse est « non », renvoi à la question n°03
EN- If the answer is "no", refer to Q°03
MG-Raha « Tsia » ny valiny, tohizana amin'ny fanontaniana n°03</td><td>◯Oui ◯Non</td></tr>
<tr><td>11-FR- Participant nouvellement diagnostiqué ?
11.EN- FR- Participant newly diagnosed?
11.MG-Mpandray anjara vaovao voatily mitondra ny raboka ?</td><td>◯Oui ◯Non</td></tr>
<tr><td>03.FR-Motif de retrait

03.EN-Reason for withdrawal from the study

03.MG-Antony nialana amin'ny fanadihadihana</td><td>◯Retrait après consentement ◯Décès ◯Perte de vue ◯Déménagement hors de la zone d'étude ◯Autres
◯Removal after consent ◯Death ◯Loss of sight ◯Move out of study area ◯Other
◯Fialana taorian'ny fanekena an-tsitrapo ◯Fahafatesana ◯Mpandray anjara tsy hita</td></tr>
</table>

<table>
<tr><td></td><td>◯Fifindramonina ivela'ny faritra misy ny fanadihadiana
◯Hafa</td></tr>
<tr><td>04.FR- Autre motif à préciser
04.EN-Other reason to specified
04.MG-Antony hafa mila faritana</td><td></td></tr>
<tr><td colspan="2">FR-En cas de décès
EN-In case of death
MG-Ho an'ny tranga fahafatesana</td></tr>
<tr><td>05.FR-Date de décès
05.EN-Date of death
05.MG-Daty nahafatesana</td><td>|__|__| / |__|__| / |__|__|__|__| (jj/mm/aaaa)
|__|__| / |__|__| / |__|__|__|__| (dd/mm/yyyy)
|__|__| / |__|__| / |__|__|__|__| (aa/vv/tttt)</td></tr>
<tr><td>06.FR-Cause de décès si conuue
06.EN-Cause of death if known
06.MG-Anton'ny fahafatesana raha fantatra</td><td></td></tr>
<tr><td colspan="2">FR- Retrait après consentement
EN-Withdrawal after consent
MG- Fialana taorian'ny fanekena an-tsitrapo</td></tr>
<tr><td>07.FR- Préciser la raison du retrait
07.EN-Specify the reason of withdrawal
07.MG-Farito ny anton'ny fialàna</td><td></td></tr>
<tr><td>08.FR-Date de signature du retrait
08EN-Date of the withdrawal signature
08.MG-Daty nanaovana sonia ny fialàna</td><td>|__|__| / |__|__| / |__|__|__|__| (jj/mm/aaaa)
|__|__| / |__|__| / |__|__|__|__| (dd/mm/yyyy)
|__|__| / |__|__| / |__|__|__|__| (aa/vv/tttt)</td></tr>
</table>

	**TB WGS cRCT Haute Matsiatra**<br>*« Séquençage du Génome Complet de la Tuberculose pour le Contrôle de la Maladie à Madagascar - Un Essai Contrôlé Randomisé en Grappe pour Évaluer Différentes Stratégies d'Intervention à l'Échelle Communautaire »*<br><br>**CAHIER D'OBSERVATION**<br>**CRF12_WGScRCT_V4_2022.09.12**

FR – Collecte des issues cliniques de l'étude
EN – Trial clinical outcome data collection
MG- Fanangonana ny vokatry ny fikarohana

**FR - IDENTIFICATION**<br>*EN - IDENTIFICATION*<br>MG -FAMANTARANA	
**01.FR-Numéro du patient dans le registre**<br>*01.EN-Patient number in the register*<br>01.MG-Laharan'ny mpandray anjara ao anaty boky	**|__|__|- |__|__|__|-|__|__|__|__|**<br>**(numéro CDT-Numéro dans le registre-année)**<br>*|__|__|- |__|__|__|-|__|__|__|__|*<br>*(CDT number-Number in th register-year)*<br>|__|__|- |__|__|__|-|__|__|__|__| (laharana CDT-Laharana ao anaty rezistra-taona)

**02.FR – Nom et prénoms**<br>*02.EN - Last and first name*<br>02.MG – Anarana sy fanampin'anarana	
**03.FR - Age**<br>*03.EN – Age*<br>03.MG – Taona<br><br>**FR-Remplir si âge supérieur à 1 an**<br>*EN-To be completed if older than 1 year*<br>MG- Fenoina raha 1 taona no miakatra	**|__|__|(année)**<br>*|__|__|(year)*<br>|__|__|(taona)
**04.FR - Age**<br>*04.EN – Age*<br>04.MG – Taona<br><br>**FR-Remplir si âge inférieur à 1 an**<br>*EN-To be completed if less than 1 year*<br>MG- Fenoina raha latsaky ny 1 taona	**|__|__|(mois)**<br>*|__|__|(month)*<br>|__|__|(volana)
**05.FR - Sexe**<br>*05.EN - Gender*<br>05.MG - Fananahana	◯**Masculin** ◯**Féminin**<br>◯*Male* ◯*Female*<br>◯Lahy ◯Vavy
**06.FR-Adresse complete**<br>*06.EN-Full address*<br>06.MG-Adiresy feno	
**07.FR - Fokontany**<br>*07.EN –Fokontany*<br>07.MG -Fokontany	
**08.FR -hameau**<br>*08.EN - village*<br>08.MG - vondrotrano	
**09.FR - Nom du CDT**<br>*09.EN - Name of CDT*<br>09.MG - Anaran'ny CDT	

**10.FR - Date de l'enregistrement dans le registre CDT**<br>*10.EN - Date of entry in the CDT register*<br>10.MG - Daty nandraisana an-tsoratra tao anaty boky CDT	**|__|__| / |__|__| / |__|__|__|__| (jj/mm/aaaa)**<br>*|__|__| / |__|__| / |__|__|__|__| (dd/mm/yyyy)* |__|__| / |__|__| / |__|__|__|__| (*aa/vv/tttt)*
**11.FR - Participant de l'étude WGS ?**<br>*11.EN -WGS Study participant ?*<br>11.MG - Mpandray anjara amin'ny fikarohana WGS ve ?<br><br>**FR-Si « non », fin de questionnaire**<br>*EN-If « no », end of questionnaire*<br>MG-Raha "tsia", mifarana ny andiam-panontaniana	◯**Oui** ◯**Non**<br>◯*Yes* ◯*No*<br>◯Eny ◯Tsia
**12.FR – Identifiant de la personne dans WGS**<br>*12.EN - Participant ID number in WGS*<br>12.MG - Raha eny,farito ny mari-pamatarana ilay olona ao anaty WGS	**PERS|__|__|__|__|__|**<br>*PERS|__|__|__|__|__|*<br>PERS|__|__|__|__|__|
